# Logistics of community smallpox control through contact tracing and ring vaccination: a stochastic network model

**DOI:** 10.1186/1471-2458-4-34

**Published:** 2004-08-06

**Authors:** Travis C Porco, Karen A Holbrook, Susan E Fernyak, Diane L Portnoy, Randy Reiter, Tomás J Aragón

**Affiliations:** 1 San Francisco Department of Public Health, Community Health and Epidemiology Section, Epidemiology and Effectiveness Research Unit, 101 Grove Street Suite 204, San Francisco, California 94102 USA; 2 Center for Infectious Disease Preparedness, School of Public Health, University of California, Berkeley, USA; 3 Surveillance and Epidemiology Section, Tuberculosis Control Branch, Division of Communicable Disease Control, California Department of Health Services, Berkeley, California, USA

## Abstract

**Background:**

Previous smallpox ring vaccination models based on contact tracing over a network suggest that ring vaccination would be effective, but have not explicitly included response logistics and limited numbers of vaccinators.

**Methods:**

We developed a continuous-time stochastic simulation of smallpox transmission, including network structure, post-exposure vaccination, vaccination of contacts of contacts, limited response capacity, heterogeneity in symptoms and infectiousness, vaccination prior to the discontinuation of routine vaccination, more rapid diagnosis due to public awareness, surveillance of asymptomatic contacts, and isolation of cases.

**Results:**

We found that even in cases of very rapidly spreading smallpox, ring vaccination (when coupled with surveillance) is sufficient in most cases to eliminate smallpox quickly, assuming that 95% of household contacts are traced, 80% of workplace or social contacts are traced, and no casual contacts are traced, and that in most cases the ability to trace 1–5 individuals per day per index case is sufficient. If smallpox is assumed to be transmitted very quickly to contacts, it may at times escape containment by ring vaccination, but could be controlled in these circumstances by mass vaccination.

**Conclusions:**

Small introductions of smallpox are likely to be easily contained by ring vaccination, provided contact tracing is feasible. Uncertainties in the nature of bioterrorist smallpox (infectiousness, vaccine efficacy) support continued planning for ring vaccination as well as mass vaccination. If initiated, ring vaccination should be conducted without delays in vaccination, should include contacts of contacts (whenever there is sufficient capacity) and should be accompanied by increased public awareness and surveillance.

## Background

Concerns about intentional releases of smallpox have prompted extensive preparations to improve our ability to detect and respond to an outbreak of smallpox [1,3,4,2]. Many factors contribute to the public health challenge of understanding and preparing for smallpox, including the age and quality of epidemiological data on native smallpox and the smallpox vaccine, the difficulty of extrapolating that data to our current populations, the possible terrorist use of altered smallpox, our ignorance of terrorist methods of release, and the relatively high risk of adverse events caused by the smallpox vaccine.

The Centers for Disease Control and Prevention (CDC) established ring vaccination (selective epidemiological control [5]), a strategy in which contacts of cases are identified and vaccinated, as the preferred control measure in the event of a smallpox outbreak (interim plan). The successful use of ring vaccination during the smallpox eradication campaign and its logical emphasis of case-contacts for immediate vaccination support its use (though the attribution of the success of the eradication program to ring vaccination has been challenged [6]). Health Officers should initiate ring vaccination upon identification of the first cases of smallpox. However, there are legitimate concerns regarding the ability of public health practitioners to mount a quick, comprehensive and successful ring vaccination program, particularly in the face of a moderate-sized or large smallpox outbreak. To guide preparation efforts and inform incident decision-making, we attempt to identify outbreak characteristics and response capacities that significantly impact the ability of ring vaccination to control a smallpox outbreak and to determine whether ring vaccination is useful in the presence of a mass vaccination campaign. Our analysis uses a newly developed mathematical model: a continuous-time, event-driven network simulation model of smallpox ring vaccination.

Mathematical models can advance our understanding of how a smallpox outbreak might progress. Several mathematical and computer models address the question of smallpox transmission [7-13]. The first model to appear [8] concluded that ring vaccination would be effective, but did not treat response logistics in detail; the model was linear and did not treat the depletion of susceptibles as the epidemic progressed (appropriate, however, for assessing control early in an epidemic, when the number infected is small compared to the number of susceptibles, e.g. [14]). The innovative model by Kaplan et al. [9] emphasized the importance of resource limitation and the logistics of smallpox response, but assumed that full infectiousness began before the onset of symptoms (and the subsequent identification and removal), and did not separately monitor close epidemiological contacts of patients (which are at greatest risk, but also easiest to find and vaccinate); the conclusions were highly critical of ring vaccination. The model by Halloran et al. [11], a stochastic, discrete-time network model omitted the explicit inclusion of response logistics while otherwise used parameter values similar to those in Kaplan et al. [9]; the inclusion of residual immunity from individuals vaccinated prior to the discontinuation of routine vaccination, however, led to a more favorable view of ring vaccination. The model by Bozzette et al. [12] assumed that ring vaccination would reduce the number of transmissions and focused on health care workers (but did not explicitly include the network structure of the population nor the response logistics of ring vaccination). The model by Eichner [15] did not explicitly include the network structure of the population nor the logistics of ring vaccination, but did use parameters based on data from an outbreak in Nigeria, and did distinguish close and casual contacts, case isolation, and surveillance of contacts; it concluded that case isolation and contact tracing could prevent the spread of smallpox. Finally, the individual-based model by Epstein et al. [16] presented scenarios illustrating certain alternatives to pure mass vaccination and ring vaccination of contacts of cases in preventing smallpox transmission in small populations of 800 individuals; this model includes no homogeneity assumptions, but did not analyze tracing of contacts of contacts.

Because none of the available models includes both network structure (with explicit contact tracing) and response logistics limited by the number of available disease control investigators [9], we included these features in a continuous-time event-driven network simulation model of smallpox ring vaccination. Specifically, the model we developed includes the following features:

### Network structure

Smallpox was primarily a disease of close contact, especially household contacts [5]. Such contacts are both the most important epidemiologically, and also the easiest to identify.

### Post-exposure vaccination

Some evidence suggests that vaccination soon after exposure may lessen the severity of the resulting case of smallpox or possibly prevent disease entirely [17-20].

### Second ring

Ring vaccination may involve not only vaccinating contacts of cases, but also contacts of contacts of cases [21,22] – potentially allowing the public health authorities to "outrun" the chain of transmission.

### Response capacity

Limited case-finding and vaccination capabilities lead to the possibility that it may be impossible to find newly exposed individuals and vaccinate them in time, resulting in a "race to trace" [9].

### Heterogeneity in natural history

Mild, ambulatory cases of smallpox may spread disease because such cases may be harder to recognize.

### Prior vaccination

Vaccination of individuals prior to the discontinuation of routine vaccination may provide some, possibly considerable, protection against infection [11,23,24], although it may also result in more mild cases which may be harder to detect.

### Public awareness

Public awareness may lead to more rapid detection of cases.

We use this model to determine what factors promote or hinder the success of ring vaccination during a smallpox outbreak, and whether ring vaccination is useful in the presence of a mass vaccination campaign. In particular, the goal of this paper is to examine the control of smallpox by contact tracing and ring vaccination using a network model which includes response logistics [9].

## Methods

### Model structure

#### Natural history of smallpox

We briefly review relevant features of the natural history and epidemiology of smallpox [17,25,8,28]. Following infection by the variola virus, individuals exhibited an incubation period of approximately 7–19 days with 10–14 being most typical. Sudden onset of fever and malaise, often with accompanying headache and backache, began the initial (or pre-eruptive) phase of smallpox. After 2–3 (or perhaps 4) days, individuals with the most common form, ordinary type smallpox, developed the characteristic *focal rash*, preceded in many cases by oropharyngeal lesions. In fatal cases of ordinary smallpox, death often occurred between the tenth and sixteenth day of symptoms; among survivors, most scabs had separated by day 22–27 of illness [26].

The course of smallpox varied widely between individuals, and several different clinical classifications were developed [29-31,17,26]. Consideration of the clinical features and severity of smallpox is important from the standpoint of mathematical transmission modeling because (1) the clinical features affect the ease of diagnosis (and thus of case identification), (2) more severe forms of smallpox may result in more transmission, (3) vaccinated individuals may develop less severe disease. We utilize a modified or simplified version of the classification system developed by Rao [32,31,26]; for the mathematical model, we will classify smallpox into five categories: early hemorrhagic, flat and late hemorrhagic, ordinary, modified, and mild. However, the clinical features and severity of smallpox in different populations may have been affected by underlying host factors, differences in viral strains, or differences in the infectious dose owing to different prevailing modes of transmission, and thus robust and precise quantitative estimates of the effects of (pre- or post-exposure) vaccination on the resulting smallpox severity, or of the infectivity differences between individuals exhibiting different forms of smallpox, are not available. The significance of such differences will be revealed through sensitivity analysis. Further details are given in Appendix 1 [see Additional file: 1].

Vaccination with vaccinia virus provided substantial protection against infection. Dixon assessed the risk of infection for an individual successfully vaccinated 3 years prior to exposure to be 0.1% the infection risk of an unvaccinated individual [17]. However, smallpox vaccination did not always take when applied, and moreover, in many instances, individuals who experienced a repeated vaccination failure developed severe smallpox upon exposure. The probability of a successful take depended on the vaccination method used; we assume that the take rate is between 95% and 100% [22,28]. In addition to protection against infection, vaccination could in many cases modify the course of infection and reduce the severity. Vaccine protection waned over time, but individuals vaccinated 20 years prior to exposure were believed to still have half the infection probability that an unvaccinated person had [17], and to have some protection against the most severe manifestations of smallpox. Dixon [17] believed that vaccine protection had at least three components, which decayed at different rates; for the purpose of this paper, we will assume that the severity of smallpox in previously any (recently or otherwise) vaccinated individuals follows the same distribution as for the vaccinated subjects seen in the case series observed by Rao in Madras [26], except that anywhere from 0 to 5% of vaccinated subjects develop smallpox too mild to diagnose without special surveillance or awareness. Observe that the vaccinated cases studied by Rao were vaccinated (at some point in their lives) before exposure, rather than after exposure to smallpox.

Smallpox was largely a disease of close contacts [17,26,33], spread primarily through face to face contact with an infected person (or occasionally through contaminated clothing). Individuals in the incubation period of smallpox were not infectious, and long term carriers did not exist. Patients were believed to be infectious following the development of oropharyngeal lesions, which could precede the rash by 24 hours [26]. However, patients were believed to be most infectious during the first week of the rash [26]; Dixon (1962) believed that patients could be infectious from the onset of acute viremia, but most evidence suggested that little transmission occurred prior to the development of the rash [26,33]. The more severe the case, the more infectious they appeared to be [34]; mild cases were believed to have very little infectiousness. While scabs contained infectious material and patients were considered to be infectious until the last scab fell off, in practice patients were not highly infectious during the scabbing phase. Importantly, patients who had been vaccinated were found to cause fewer secondary cases [34]. Very severe cases, such as hemorrhagic or flat smallpox, occasionally resulted in considerable transmission, owing to diagnostic difficulties; mild cases, in which the patient remained ambulant during the course of the disease, could cause considerable spread as well [35,36]. Within a household or family dwelling, the secondary attack rate of unvaccinated susceptibles depended on the time and place, occasionally below 50% [29], but often approaching 100% [37]. Drier conditions were often believed to favor transmission [17,27], so that lower rates of transmission derived from tropical regions may not be applicable to the temperate zone [38]. The number of secondary cases resulting from a given importation into Europe varied widely [39], with most importations yielding few cases, but with the occasional large outbreak being seen.

**Figure 1:**
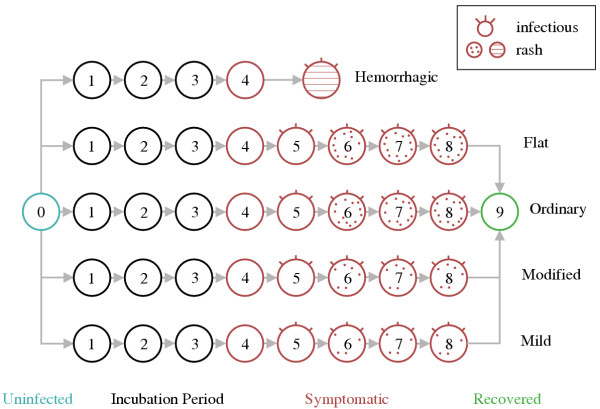
**Smallpox stages used in the simulation model. **Flat and ordinary smallpox rashes are indicated with more dots than modified and "mild" smallpox, suggesting potentially greater infectiousness. Hemorrhagic smallpox is indicated by horizontal line shading. Further details are provided in Table 6.

Mathematically, we represent the course of smallpox according to Figure 1. We distinguish eight epidemiologically relevant states: (1) just following exposure, during which time vaccination could afford complete protection against disease, (2) a period of several days during which vaccination will not prevent disease, but may still reduce the severity of disease, (3) still prior to the development of symptoms, but too late for vaccination, (4) the beginning of the pre-eruptive period, during which the patient exhibits fever, malaise, and possibly other symptoms, but is not yet infectious, (5) a short period prior to the appearance of the rash, during which the appearance of oropharyngeal lesions will permit variola transmission, (6) the first week of the rash, during which time the patient is most infectious, (7) and (8), succeeding stages of the rash, during which time the patient is less infectious. For each of these states, we assume that conditional on surviving, the waiting time until the next stage is chosen from a uniform distribution as indicated in Appendix 2 [see Additional file: 2], except that the incubation period (the time from infection until Stage 4) is derived from estimates of the incubation distribution of smallpox based on importation cases in Europe [26] (see Appendix 2 [see additional file 2] for details). We chose to sample from a uniform distribution as a simple way to ensure a minimum waiting time in each state; many alternatives to this choice are possible.

#### Network structure

We simulate the transmission of smallpox on a "small-worlds" network (highly clustered, but with short characteristic path lengths) [40]. Specifically, we assume that each person is located in a single household, and that the transmission rates were greatest in the household. We also assume that a fraction of the population are grouped into workplace or social groups, in which transmission may also occur, but with a lower rate per unit time than for household contacts. Finally, we assume that with a still smaller probability, any individual may transmit infection to any other individual in the population (casual contacts).

**Figure 5:**
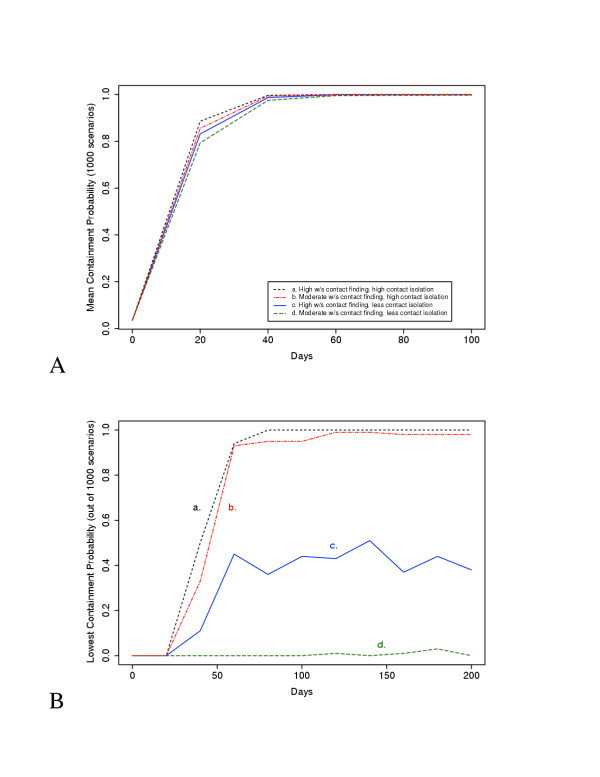
**5A - The mean containment probability **increases as the number of ring vaccinations per day is increased. For this figure, the 1000 "calibrated" parameter sets were chosen, and for each parameter set, 100 realizations were simulated and the fraction of these for which the epidemic was contained to fewer than 500 cases was determined. The average of these 1000 containment fractions is plotted on the vertical axis. We assumed a household contact finding probability of 95% and that the diagnosis rates double after community awareness of the epidemic. We considered high levels of workplace/social (w/s) contact finding (0.9), as well as moderate levels (0.8). We also considered two levels of diagnosis of smallpox among investigated (alerted) contacts: high levels (corresponding to a 3 hour mean delay, indicated by "high contact isolation"), and moderate levels (corresponding to a one day delay, and indicated by "less contact isolation"). The figure shows four such conditions, **a**. high workplace/social contact finding probability and high contact isolation, **b**. moderate workplace/social contact finding probability and high contact isolation, **c**. high workplace/social contact finding probability and less contact isolation, and **d**. moderate workplace/social contact finding probability and less contact isolation. All other parameter values were chosen from the uncertainty analysis (the 1000 "calibrated" parameter sets). In this figure, "contact isolation" refers to the monitored diagnosis rate, i.e. the rate at which previously asymptomatic contacts who subsequently develop disease will be diagnosed (φ, Table 1, Table 8). **5B - The minimum containment probability **out of the same 1000 scenarios chosen in Figure 5A. Whereas in Figure 5A, we averaged the simulated containment frequency (out of 100 realizations for each scenario), in this figure we determined which of the 1000 scenarios led to the lowest containment frequency, and we plotted this single worst (out of 1000) containment frequency, at different levels of ring vaccination capacity, for the same four conditions as in Figure 5A: **a**. high workplace/social contact finding probability (0.9) and high contact isolation (effective 3 hour delay following symptoms), **b**. moderate workplace/social contact finding probability (0.8) and high contact isolation, **c**. high workplace/social contact finding probability (0.9) and less contact isolation (effective one day delay), and **d**. moderate workplace/social contact finding probability (0.8) and less contact isolation. All parameters are the same as in Figure 5A (the household contact finding probability is 0.95 for all scenarios, and the diagnosis rates are doubled after the onset of community awareness). In this figure, "contact isolation" refers to the monitored diagnosis rate, i.e. the rate at which previously asymptomatic contacts who subsequently develop disease will be diagnosed (φ, Table 1, Table 8).

In general, in a network-structured model, the number of secondary cases caused by an index case in a completely susceptible population is not a useful index of epidemic potential [41,42] (for a simple example, see [43]), since (for instance) an individual could infect everyone in his or her household, and not cause a widespread epidemic unless between-household transmission were sufficiently frequent. Rather than constructing the appropriate generalized basic reproduction number for our model (leading to highly cumbersome expressions), we chose an alternative (ad hoc) index of epidemic potential. For any given scenario of interest, we simulated the introduction of 10 index cases at random into a population of size 10000, and operationally defined "containment" to occur whenever the final size of the epidemic was less than 500 cases within 250 days (we showed, in the discussion of Figure 5A below, that in nearly all cases, the 250-day window differs very little from a 1000-day window). Because we simulate a disease with a finite duration on a finite and non-renewing population, epidemic extinction always occurs in finite time.

#### Medical and public health intervention

We assume that even in the absence of specific case investigations, the presence of smallpox symptoms will prompt patients to be diagnosed; we assume, however, a higher diagnosis rate for all forms of ordinary smallpox than for the severe flat and hemorrhagic forms, or for the mildest form. We assume that once an individual is diagnosed, their household and workplace contacts are investigated and detected with some probability; we assume that a high fraction (such as 95%) of household contacts are assumed to be traceable (see below). We assume that the fraction of workplace/social contacts that are traceable is less than the fraction of household contacts that are traceable; we assume that no casual contacts are traceable.

High contact-finding rates may be plausible; we examined San Francisco Department of Public Health records of contact investigations for meningococcal disease (like smallpox, a potentially fatal disease for which rapid intervention may prevent mortality and morbidity). Records were available from December 2001 to April 2002; 13 such investigations during this period resulted in identification of 62 household contacts, all of which were contacted; out of 38 workplace/social contacts identified, 32 were contacted (84%).

In our model, we assume that identified asymptomatic contacts are vaccinated, quarantined, and monitored for symptom development, while symptomatic patients are isolated and treated as necessary [9]; thus, the modeled interventions include more than ring vaccination alone. Finally, we include the possibility that all contacts (of both symptomatic and asymptomatic) traced and the same procedure applied, so that all contacts of contacts would be investigated. We assume that uninfected or asymptomatic individuals who are visited or traced individuals will be diagnosed more rapidly than if they had not been traced; in fact, such individuals would be isolated and would not be able to continue a chain of transmission. We follow previous models [9] in assuming a limited vaccination capability of *K*_*r *_per day for ring vaccination. We assumed one of two strategies for contact tracing: (1) tracing only of direct contacts of diagnosed cases, and (2) tracing of contacts of contacts of diagnosed cases as well as direct contacts.

**Figure 2:**
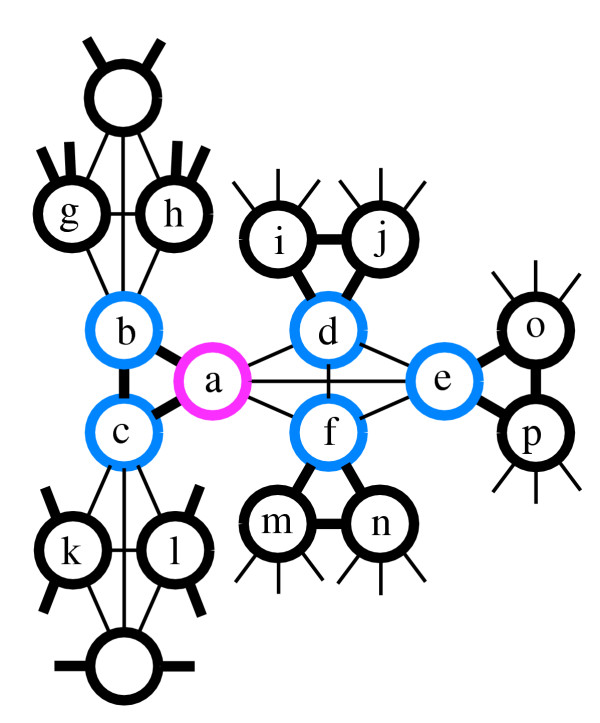
**Network structure **shown for households (joined by thick lines) of size 3 and workplace/social groups of size 4 (joined by thin lines); a small portion of the network is shown. Individual *a *has two household contacts (*b *and *c*), and three workplace/social contacts (*d*, *e*, and *f*). If individual *a *were a smallpox case, the household contacts would be at highest risk for acquiring smallpox, followed by workplace/social contacts; all individuals in the population are at a low risk of casual transmission from individual *a*. Case investigation of individual *a *would identify the direct contacts *b-f *with probabilities that depend on whether the contact is household or workplace/social; if such individuals are identified, they will be vaccinated. If contacts of contacts are being traced, the investigation will subsequently identify individuals *g-p*.

The contact structure of the network is illustrated in Figure 2. Observe that individuals *b *and *c *are household contacts of individual *a*, so that if individual *a *were a smallpox case, an attempt would be made to find and vaccinate individuals *b *and *c *as household contacts of a case. If individuals *a *and *b *were both cases, then two attempts could be made to find individual *c*. We have modeled the effect of multiple contact-finding attempts conservatively in the sense that if the first attempt to find an individual as a household contact (of a case or of a contact) is determined to fail, no further attempts will be made. This maintains the failure rate of contact tracing (looked at from the standpoint of finding individuals) even in large households. Similar considerations apply to workplace/social groups.

### Analysis

We analyzed the model in three ways. First, we selected a Latin Hypercube sample [44-46] of parameters chosen uniformly from the parameter ranges given in Appendix 2 [see additional file 2], and simulated the transmission and control of smallpox to determine which parameters were most important for contact tracing and ring vaccination to be effective. Second, we used the same Latin Hypercube Sample of input parameters, but assumed that all disease control efforts were inactive. We used these parameters to simulate smallpox transmission, but then iteratively selected transmission parameters so that (1) between 1% and 10% of new infections resulted from casual (random) transmission, and (2) each index case resulted in between two and five secondary cases (thought to be plausible for historic smallpox; [8] suggest three secondary cases). For each of the resulting smallpox parameter sets using 100 stochastic simulations per set, we determined the daily ring vaccination/case tracking capacity needed to contain all simulated smallpox epidemics (i.e., keep the total number of cases below 500 within 250 days). Third and finally, we chose parameter values to yield an moderately large smallpox epidemic (with each index case causing approximately six secondary cases), and present illustrative scenarios for ring vaccination. These scenarios are intended to complement the simulations which were calibrated to historic smallpox, since the characteristics of smallpox that may be used in a deliberate release are not known. It is important to realize that in our model, the case finding time determines the fraction of contacts that will become infected, and that our model parameters have been chosen to yield quite rapid transmission to close contacts; in reality, much transmission of natural smallpox occurred through "sickbed" routes which would not occur in a modern setting [47], so that in this sense our model errs considerably on the side of caution and pessimism.

## Results

### Most important parameters (sensitivity analysis)

To determine which of the input parameters were most important in determining the total number of smallpox cases, we selected a Latin Hypercube sample of size 1000 from the input parameter ranges indicated in Appendix 2 [see additional file 2] and simulated the mean number of cases within 250 days in a population of 10000. We then computed the partial rank correlation coefficient [46] (PRCC; see Appendix 2 [see additional file 2]) between each input parameter and the number of smallpox cases; when the PRCC is close to zero, the value of the parameter has little relation to the simulation output; when the PRCC is close to +1.0 or -1.0, the value of the parameter is highly important in determining the simulation output. Neglecting the number of index cases (which is directly related to the number of new cases), those parameters whose PRCC exceeds 0.1 are shown in Table 2. Most of these parameters identified as important are related to the density of available contacts (mean household size, prior vaccination fraction, and protection due to prior vaccination) or the transmission rate and infectivity (including the length of the pre-eruptive infectious period (stage 5 in Figure 2)). Note, however, that the speed of ring vaccination (household tracing delay) and faster diagnosis due to awareness of the outbreak are important parameters. Additionally, the infectivity of mild cases appears as an important parameter as well.

**Table 2:** Most important parameters. PRCC: partial rank correlation coefficient (see Appendix 2 [see additional file 2] for definition and references).

Parameter	PRCC
Mean Household Size	0.575
Transmission Rate from Close Contacts	0.520
Infectivity prior to rash	0.309
Ring Vaccination Capacity	-0.296
Casual Transmission Probability	0.244
Pre-eruptive infectious period (lower bound)	0.224
Number of Casual Contacts per Day	0.210
Relative Infectiousness of Social/Workplace Contacts	0.200
Fraction of Individuals in Social/Workplace Groups	0.183
Faster Diagnosis due to Awareness of Outbreak	-0.175
Household Tracing Delay	0.104
Pre-eruptive Diagnosis Probability	-0.103
Diagnosis Probability after Rash	-0.103

### Illustrative scenarios

To explore factors which contribute to the success of ring vaccination, we chose smallpox scenarios which resulted in severe and fast-moving epidemics in the absence of disease control; these simulated epidemics are considerably more severe than is believed likely under present circumstances.

#### Effect of contact tracing and ring vaccination

**Figure 3:**
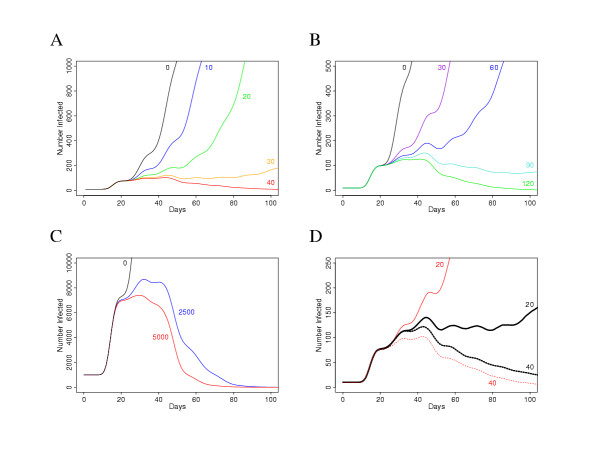
**3A - Expanding severe smallpox epidemic **beginning with 10 initial cases, assuming 0, 10, 20, 30, and 40 possible ring vaccinations per day. The household size is 4 and the workplace/social group size is 8; we assume 95% of household contacts are traceable (with a mean delay of 1 day) and 80% of workplace/social contacts are traceable (with a mean delay of 2 days). We also assume that 25% of the population have 50% protection from infection resulting from vaccination prior to the discontinuation of routine vaccination. We assume that infection will be transmitted to close contacts with a mean time of 0.2 days, and that each person while infective causes on average 0.15 casual (untraceable) infections per day. We assume that individuals are 20% as infectious in the day just before the appearance of the rash as they will be during the first week of the rash, and that individuals are 20% as infectious as this (4% as infectious as during the first week of the rash) during the prodromal period. We assume that diagnosis rates will increase by a factor of 50% after smallpox becomes known to the community; we assume that each individual contacted during an investigation has a additional diagnosis or removal rate of 0.75 per day following the onset of symptoms (reflecting enhanced surveillance or contact isolation). Important parameters are summarized in Table 1; the full set of parameter choices is outlined in Tables 8-11 in Appendix 2 [see additional file 2]. Diagnosis times are discussed in Appendix 2 [see additional file 2]. **3B - An expanding severe smallpox epidemic under inadequate ring vaccination **is shown for parameters identical to Figure 3A, except that workplace/social group sizes are 12 (instead of 8), and the probability of tracing workplace/social contacts is 0.6 (instead of 0.8). **3C - A severe smallpox epidemic is controlled **by ring vaccination despite the large number of initial cases. The parameters are identical to Figure 3A, except that 1000 index cases inaugurate the attack in these scenarios (and ring vaccination capacity is much greater, as indicated). While not recommended, ring vaccination may ultimately halt epidemics beginning with many index cases if sufficient vaccination capacity were available, contact finding feasible, and follow-up sufficient. **3D - Tracing contacts of contacts **(red) is beneficial when sufficient contact tracing/ring vaccination capacity exists (dotted lines). In these scenarios, all parameters are the same as in Figure 3A; the number of contact tracings possible per day is either 20 or 40 per day. Contacts of contacts are traced in two scenarios; in the other two, only direct contacts of cases are traced. For low levels of ring vaccination (20 per day), tracing contacts of contacts is harmful; for high levels (40 per day) of ring vaccination, it is beneficial to trace contacts of contacts. When the contact tracing/ring vaccination capacity is too small to adequately cover contacts of the cases themselves, diversion of resources to contacts of contacts is harmful; however, provided that sufficient capacity exists, tracing contacts of contacts helps outrun the chain of transmission. Each line corresponds to the average of 100 realizations.

We used these parameters to simulate smallpox epidemics beginning with 10 cases, for a variety of levels of ring vaccination capacity per day (contact tracing capacity per day), as shown in Figure 3A. In this Figure, we assume that the population size is 10000, and that the epidemic began with 10 infected individuals. The mean household size is assumed to be 4, the mean size of the workplace/social contact group is 8, and contacts of contacts are traced. We assume that each day, the number of contacts that can be traced and vaccinated as a result of case investigation is 0, 10, 20, 30 and 40 per day; the probability of finding a workplace/social contact is assumed to be 80%. The Figure shows the average number of infected individuals each day (based on 100 stochastic simulations) for each of these scenarios. Selected parameter values are indicated in the caption for Figure 3A and in Table 1.

**Table 1:** Selected parameter values for Figure 3A and other illustrative scenarios. The notations "Other" or "Other factors" in the column "See also" refers to the text section "Other factors". The symbols are defined in Appendix 2 [see additional file 2] and are included for reference.

Description	Values	See also	Symbol
Number of index cases	10–1000	Figure 3C	*A*
Mean household size	4		*H*
Workplace/social group size	8	Figure 3B	*W*
Ring vaccinations per day	0–200	Fig. 3A, 3B, Other	*K*_*r*_
Monitored diagnosis rate	1–8 day^-1^	Figure 5A, 5B	φ
Prob. of finding household contact	0.95	Table 4	υ_1_
Prob. of finding workplace/social contact	0.8	Fig. 3B, 4AB; Tb. 4	υ_2_
Delay, tracing household contacts	1–5 days	Figure 6	δ_1_
Delay, tracing workplace/social contacts	2–10 days	Figure 6	δ_2_
Relative diagnosis rate after 1st diagnosed case	1.5	Figure 7	*a*_1_
Infectivity, stage 4 relative to stage 5	0.2	Figure 8	*k*
Infectivity, stage 5 relative to stage 6	0.2	Figure 8	*k*'
Infection hazard for close contacts	5 day^-1^	Table 3	λ
Relative hazard for workplace/social contacts	1/3	Table 3	*h*_2_
Casual transmission rate	0.15 day^-1^		β
Prior vaccination fraction	0.25	Other factors	*f*
Fraction of mild cases	0.03	Other factors	
Vaccine success rate (for very recent vaccination)	0.667	Other factors	α_1_
Vaccine success rate (vaccination prior to discontinuation of routine vaccination)	0.5	Other factors	α_2_
Vaccine success rate full protection	0.999	Other factors	α_3_

Because we assumed nonzero diagnosis probabilities during the prodromal period for all individuals in Figure 3A, we repeated the simulation assuming no diagnosis in the prodromal period unless individuals were under specific surveillance. The results were nearly identical: assuming 30 contact tracings (ring vaccinations) per day, we found 26% of the scenarios in Figure 3A exhibited decontainment, and 28% assuming no diagnosis during the prodromal period; assuming 40 contact tracings per day, we found 1 out of 100 scenarios showed loss of containment in Figure 3A and when we repeated the scenario of Figure 3A assuming no diagnosis during the prodromal period.

In Figure 3B, we illustrate control of an epidemic for which all parameters are identical to Figure 3A, except that the workplace/social group size is 12 (instead of 8, as in Figure 3A), and the probability of finding workplace/social contacts is 60% (instead of 80%, as in Figure 3A). In this case, the larger size of the workplace/social groups and the lower contact finding probability makes it necessary to have a higher ring vaccination capacity to attain a high probability of containing the epidemic, and on average it takes longer for eradication to finally occur.

Finally, in Figure 3C, we show control of an epidemic in a population of 100,000, beginning with 1000 initial infectives, keeping all other parameters the same as in Figure 3A. Each curve corresponds to the indicated number of possible ring vaccinations per day. This figure shows that assuming sufficient capacity, ring vaccination is in principle capable of containing even epidemics beginning with very many infected individuals. However, mass vaccination in such cases is justified because of the far larger number of individuals at risk and the inability to perform such extensive contact tracing.

In Figure 3D, we compare the effect of tracing contacts of contacts (as described in Appendix 2 [see additional file 2]) at different levels of ring vaccination capacity. Thin lines in red correspond to the presence of tracing contacts of contacts; thick lines in black correspond to tracing direct contacts of cases only. Each simulation was performed 100 times, with 10 initial infectives, and for 20 and 40 ring vaccinations possible per day (as indicated). The average number infected on each day is plotted in the Figure. The figure illustrates that when ring vaccination capacity is low, tracing contacts of contacts (as modeled) yields a more severe average epidemic; when ring vaccination capacity is large, tracing contacts of contacts results in a less severe average epidemic; if the contact tracing/ring vaccination capacity is too low to cover adequately the contacts of contacts in addition to the contacts of cases, extension of tracing to the contacts of contacts (the second ring) is harmful; however, if there is sufficient capacity to cover the contacts of contacts, then the tracing of contacts of contacts is beneficial.

**Figure 4:**
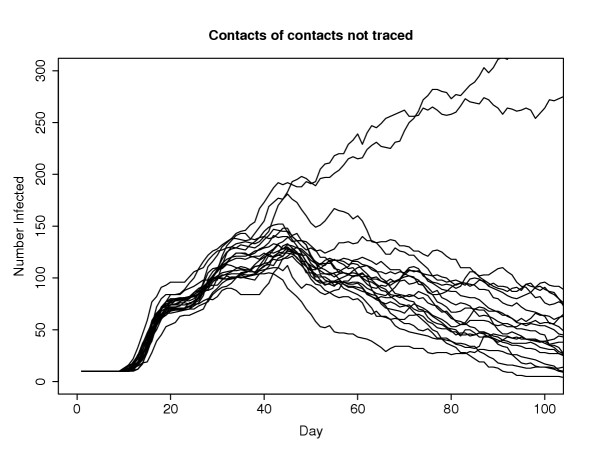
**Stochastic variability **is illustrated by plotting the number of infectives over time over multiple replications. In this example, most simulations exhibit rapid containment of smallpox. The mean number of cases (averaging over simulations) is influenced by a small number of simulations exhibiting an uncontained epidemic. The parameters are the same as in Figure 3A, except that contacts of contacts are not traced in these replications.

Finally, in Figure 4, we illustrate the considerable variability that may be seen from simulation to simulation. This figure shows twenty simulations when contacts of contacts are not traced. Stochastic variability between realizations is considerable, even when all parameters are held constant; this variability is expected to limit the ability to make inferences based on observation of a single realization of the process.

Because our baseline hazard for infection of individuals may be larger than would be expected for naturally occurring smallpox, we examined the effect of more realistic values of this hazard. In particular, we chose different levels of ring vaccination capacity (10, and 20), and of the relative hazard for workplace/social contacts, and then chose values of the baseline hazard for infection varying from 0.5 per day (for a mean time to infection of 2 days) to 2 per day (for a mean time to infection of one half day), and introduced 10 index cases into a population of 10000. We then repeated this 100 times, and reported the fraction of scenarios in which the number of infections ultimately exceeded 500 (as before, chosen as a cutoff to indicate the ultimate "escape" of containment of the epidemic). These results, shown in Table 3, support the idea that ring vaccination can easily control introduced smallpox provided there is sufficient capacity and efficacy of tracing.

**Table 3:** Estimated decontainment probability  for different levels of ring vaccination capacity (*K*_*r*_) and relative hazard for infection due to workplace/social contacts (*h*_2_), for different levels of the baseline hazard for infection from household contacts λ (based on replications of 100 simulations for each level). For each scenario, 10 index cases were introduced into a population of size 10000. All other parameters were the same as for Figure 3A. As before, we define decontainment to mean that the total number of cases from 10 index cases eventually exceeded 500 by day 250.

Relative hazard for workplace or social contacts	Ring vaccinations per day			
	10		20	
	λ		λ	
		
1/3	0.5	0	0.5	0
	0.75	0.02	0.75	0
	1	0.26	1	0
	1.25	0.73	1.25	0
	1.5	0.96	1.5	0.02
	2	1	2	0.16
	
	λ		λ	
		
2/3	0.5	0	0.5	0
	0.75	0.46	0.75	0
	1	0.82	1	0.02
	1.25	1	1.25	0.11
	1.5	1	1.5	0.26
	2	1	2	0.49
	
	λ		λ	
		
1	0.5	0.14	0.5	0
	0.75	0.86	0.75	0
	1	0.99	1	0.07
	1.25	1	1.25	0.22
	1.5	1	1.5	0.49
	2	1	2	0.85

Because of considerable uncertainty in the model parameters, we chose a collection of parameter values, and for each, estimated the containment probability (operationally defined as fewer than 500 total cases as a result of 10 index cases, within 250 days). We estimated this containment probability by simulating the smallpox epidemic 100 times for the same parameter values, and computing the frequency out of these 100 realizations for which fewer than 500 index cases resulted within 250 days. (Using a 1000 day window produces slightly smaller containment estimates; for 3 out of 1000 parameter set choices, this difference was greater than 0.06; the maximum difference seen was 0.23; the mean absolute difference was 0.0029; in only one case out of 1000 did we see containment in all 100 cases for the 250-day window, but not in all 100 cases for the 1000-day window).

One thousand scenarios chosen from a Latin Hypercube sample were analyzed, and as indicated before, we chose the hazard for close contact transmission and the hazard for random transmission to guarantee that between 2 and 5 secondary cases per case occur, and that no more than 5% of cases are attributable to random transmission (we refer to this set as the "calibrated" scenarios further in this text). Having chosen this collection of 1000 parameter sets, we considered two levels of two different control parameters which were applied to each (so that each of the 1000 parameter sets were simulated under four different control conditions). The first of the two control parameters was the probability of workplace/social group contact finding; we chose values of 0.8 and 0.9 for this parameter (the household contact finding probability was 0.95 in all cases). The second of the control parameters was the rate of diagnosis (and effective removal) from the community of cases developing among previously identified and traced contacts who were initially asymptomatic (we refer to this as the monitored diagnosis rate); we assumed first a low level corresponding to a mean diagnosis time of one day from the onset of symptoms, and a high level corresponding to a mean time of 3 hours from the onset of symptoms (high levels of the monitored diagnosis rate correspond effectively to isolation of contacts). Finally, we assumed a doubling of the diagnosis rate after the beginning of widespread community awareness of smallpox. We then computed the containment fraction at different levels of ring vaccination capacity (contact tracing capacity per day). Thus, for each of 1000 scenarios (parameter set choices), we assigned the workplace/social group contact tracing success probability (υ_2_), the monitored diagnosis rate φ (Appendix 2 [see additional file 2]), and the contact tracing/ring vaccination capacity per day (*K*_*r*_). We then performed 100 realizations beginning with 10 index cases, and computed the containment fraction (fraction showing fewer than 500 cases in 250 days, beginning with 10 index cases). Thus, for each of the two choices each of υ_2 _and φ, and for each value of *K*_*r *_we examined, we obtained 1000 values of the containment fraction. We use the resulting distributions in Figure 5A (averaging over these 1000 containment fractions), and Figure 5B (displaying the minimum value of the 1000 containment fractions).

In Figure 5A, we plot the mean containment fraction (averaging the containment fraction over all 1000 scenarios), as ring vaccination capacity varies, for the two levels of workplace/social group contact finding probabilities (0.8 and 0.9), and for the two levels of monitored diagnosis rate among initially asymptomatic contacts (1 day^-1 ^and 8 day^-1^). For low levels of ring vaccination (traceable contacts per day), the epidemic is almost never contained, but for ring vaccination levels near 50–60 per day (5–6 per index case per day), the average containment fraction became close to 1. However, this average conceals the fact that for some scenarios (parameter sets chosen from the calibrated uncertainty analysis), control remains difficult or impossible even at high levels of ring vaccination. Therefore, in Figure 5B, we plotted the single lowest containment fraction seen out of the 1000 computed; focusing on the single worst scenarios reveals a different picture, and shows that isolation of asymptomatic contacts and very high probabilities of finding workplace or social contacts would be needed to control smallpox under these most pessimistic parameter choices.

#### Effect of contact tracing speed

**Figure 6:**
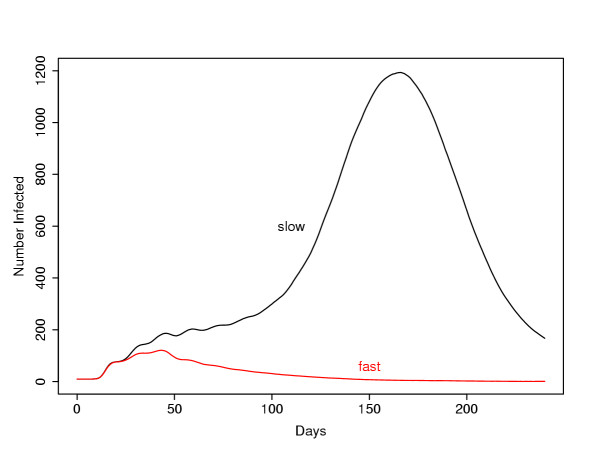
**Faster contact tracing **may improve the efficacy of ring vaccination. We assume the same baseline parameters as in Figure 3A (e.g. households of size 4, workplace/social groups of size 8, 95% of household contacts traceable, 80% of workplace/social contacts traceable), and 30 ring vaccinations available per day (with contacts of contacts not traced). The *fast *scenario corresponds to an average one day delay for household and two days for workplace/social contacts (as in Figure 3A); the *slow *scenario corresponds to an average five day delay for household and ten day delay for workplace/social contacts. This figure shows the average of one hundred realizations starting with ten index cases.

Rapid contact tracing in ring vaccination may play an important role in suppressing the epidemic, since the longer it takes to trace a contact, the less likely the vaccine is to be efficacious, and the more opportunities the infected individual may have to transmit disease before they are finally located, isolated, and vaccinated if appropriate. We illustrate this possibility in Figure 6 by examining the same scenario we showed earlier in Figure 3A (e.g. households of size 4, workplace/social groups of size 8, 95% of household contacts traceable, 80% of workplace/social groups traceable, an average time to infection for a household contact of an infective given by 0.2 days). We assume in one case that contacts may be traced quickly (1 day for a household contact, 2 days for a workplace/social contact), and in the other that the contacts are on average found slowly (5 days for a household contact, 10 days for workplace/social contacts); we assumed 30 ring vaccinations (traceable contacts) possible per day. In this scenario, the epidemic is more severe and containment (as we have been defining it) less likely when contact tracing is slow: in the *fast *scenario, 238 infections occurred on average and the (estimated) containment probability was 99%; for the *slow *scenario, on average 3587 infections occurred and the (estimated) containment probability was only 1%.

While Figure 6 illustrates the possibility that rapid contact tracing may be of decisive importance in some scenarios (parameter set choices), this is not always the case. For some parameter sets, the probability of tracing contacts (household or workplace/social) may be too low, or the transmission rate too high, for more rapid contact tracing to make any difference. Conversely, for other parameter sets, the smallpox transmission rate may be so low that smallpox is easily contained even with slow contact tracing. While rapid contact tracing is never harmful, overall, how typical are the results of Figure 6 (in which rapid contact tracing was important in ensuring the efficacy of ring vaccination)? To address this question, we simulated the growth of smallpox for the 1000 "calibrated" scenarios we used in Figure 5A and 5B. As before, we assumed ten initial cases, and (as in Figure 6) that 30 ring vaccinations were possible per day; then we simulated 100 epidemics assuming one day to find a household contact (and 2 days to find a workplace/social contact). We then simulated 100 epidemics assuming that it takes five days to find a household contact and 10 days to find a workplace/social contact (as in Figure 6). For each of these 1000 scenarios, we calculated the fraction of simulations for which the total number of cases is 500 or less within 250 days, i.e. the containment fraction. For nearly all scenarios (parameter set choices), the containment fraction was smaller (sometimes much smaller) when the contact finding time is faster (since faster contact finding, all else being equal, improves smallpox control, as illustrated in Figure 6). However, for 64.5% of the scenarios (parameter set choices) examined, the difference was less than 2.5% in absolute terms (smallpox was either contained or not contained depending on other factors, and rapid contact tracing did not make the difference). On the other hand, for 18.7% of the scenarios examined, the absolute difference in the containment probability was 20% or more; thus, a substantial difference in containment probability is occasionally attributable to the difference between fast and slow contact tracing.

#### Effect of more rapid diagnosis

**Figure 7:**
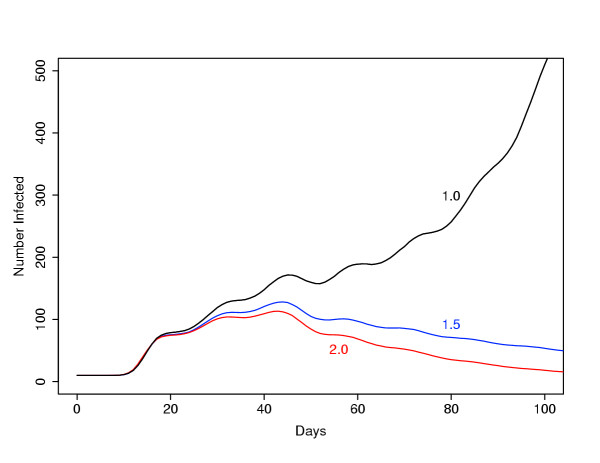
**More rapid diagnosis **due to public awareness or increased surveillance may lead to far more effective epidemic control. We assume the same baseline parameters as in Figure 3A, and averaged 100 realizations of the epidemic beginning with 10 index cases and assumed a ring vaccination capacity of 20 per day (and contacts of contacts not traced). For the black line, the diagnosis rate of cases does not change after the first case is identified (the multiplier is 1.0); for the blue line, the diagnosis rate increases by 50% (multiplier 1.5) after the first case is identified (as in Figure 3A), resulting in substantially fewer cases; and for the red line, the diagnosis rate is doubled (multiplier 2.0) after the first case is identified, resulting in still fewer cases.

Public awareness of smallpox, leading to more rapid isolation and identification, may play an important role in eliminating the epidemic, as illustrated by the scenarios in Figure 7. We assumed 20 ring vaccinations possible per day, a capacity too small to contain the epidemic in the absence of increased surveillance or diagnosis; the black line in the figure shows the steeply rising average number of cases for the first 100 days. If, however, surveillance or public awareness of the symptoms of smallpox increases the diagnosis rate by 50% (multiplies the baseline diagnosis rates by 1.5), containment becomes possible (blue line); with a doubling of the diagnosis rate (red line) the peak number of cases is lower still. In these scenarios, increased diagnostic rates markedly improve the ability of ring vaccination to control the epidemic, this suggest that any ring vaccination effort be accompanied by increased public awareness and surveillance.

In many cases, however, more rapid diagnosis was not required for ring vaccination to be effective. As before, we simulated smallpox epidemics for each of 1000 calibrated scenarios, performing 100 realizations each beginning with 10 index cases, and computed the fraction of scenarios for which the epidemic was always contained (as defined earlier), assuming no change in diagnosis rates. We assumed 80 ring vaccinators per day, contact finding probabilities of 0.95 for households and 0.8 for workplace/social contacts (as in Figure 3A). Under these assumptions, for 83.4% of the scenarios, the epidemic was contained within 500 total cases in each of the 100 realizations, even with no change in diagnosis rates. Uncertainty analysis (using the 1000 calibrated scenarios, and based on the fraction of 100 replications showing decontainment) revealed the most important parameters which predict the failure of ring vaccination without more rapid diagnosis were the same as we found in the earlier uncertainty analysis; a higher fraction vaccinated before the epidemic, smaller households or workplace/social groups, less transmissibility, lowered infectivity prior to the rash, more rapid diagnosis, and a higher rate of diagnosis for alerted individuals all contribute to a greater containment probability even without an overall increase in the diagnosis rate.

#### Effect of continued surveillance of contacts

We have been assuming that whenever an individual is contacted during an investigation, the individual will be diagnosed more quickly should they subsequently develop symptoms. When transmission is assumed to be very rapid (smallpox is assumed to be highly contagious), most individuals may already be infected when identified through contact tracing from an infective. Using the scenario we examined in Figure 3A, we see that continued surveillance of contacts is an essential component of effective ring vaccination designed to control rapidly spreading smallpox: if smallpox in a contact is not diagnosed any more quickly than for a non-contact, containment by ring vaccination requires over 98% contact finding probabilities for both household and workplace/social contacts – even if unlimited numbers of ring vaccinators are available; containment cannot be guaranteed by adding additional ring vaccination capacity if the contact finding rates are too low and/or the follow-up for contacts is insufficient. Smallpox which is transmitted less rapidly to contacts would, however, be containable with a lower contact finding probability (results not shown).

Finally, we used the "calibrated" scenarios (parameter set choices) to explore the levels of contact finding probability needed to contain the epidemic (as before, defined to mean 500 or fewer cases ultimately resulting from ten initial cases) (Table 4). In these scenarios, we assumed that all traceable contacts were followed up very quickly (1/*a *= 1 hour, so that cases arising in previously contacted persons almost never transmit the infection further). We chose different levels of household and workplace/social contact finding probabilities and different levels of ring vaccination capacity, and performed 100 replications of each of the 1000 different scenarios. In Table 4 we report the fraction of scenarios for which all 100 replications exhibited containment. Scenarios in which smallpox is highly contagious require high contact finding probability to ensure the containment of the epidemic.

**Table 4:** Containment of severe smallpox at different levels of contact finding. The first three columns are assumed levels for the probability of finding a household contact, the probability of finding a workplace/social (W/S) contact, and for the number of contact tracings/ring vaccinations possible per day; the last two columns express (as percentages) the resulting probability of containment given the assumed contact finding probabilities and contact tracing capacities; two containment probabilities are given: the containment probability when only contacts of cases are traced (first column, "Contacts"), and the containment probability when contacts of contacts of cases are traced in addition to the contacts of cases (second column, "Contacts of Contacts"). All other parameters are the same as in Figure 3A.

Probability of finding		Number of Ring vacc. per day	Containment Contacts	Probability when Tracing Contacts of contacts
Household contacts	W/S contacts			
0.95	0.85	50	99.1%	97.9%
0.95	0.85	100	99.3%	100.0%
0.95	0.85	200	99.1%	100.0%
0.9	0.8	50	95.7%	95.8%
0.9	0.8	100	95.6%	99.9%
0.9	0.8	200	95.4%	100.0%
0.85	0.75	50	86.0%	93.3%
0.85	0.75	100	86.1%	99.1%
0.85	0.75	200	86.3%	99.2%
0.75	0.6	50	52.1%	72.0%
0.75	0.6	100	51.5%	78.5%
0.75	0.6	200	53.0%	78.6%

#### Transmission prior to rash

**Figure 8:**
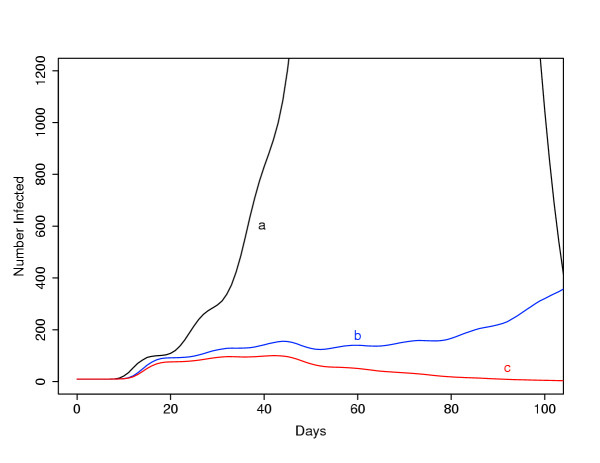
**Transmission prior to the rash **makes epidemic control more difficult. The figure shows a expanding smallpox epidemic assuming differing levels of infectivity prior to the rash. We assume all parameters are the same as in Figure 3A (and that the ring vaccination capacity is 40 per day). Infectivity prior to the rash is modeled as the relative infectivity during the short (1 day) period of oropharyngeal lesions just prior to the rash (compared to the infectivity during the first week of the rash), and as the relative infectivity during the prodromal period (relative to the period just prior to the rash). For scenario **a**, relative infectivity during the prodromal period and just prior to the rash is the same as during the first week of the rash, for scenario **b**, the relative infectivity just prior to the rash is the same as during the first week of the rash, but during the prodromal period is 4% (as in Figure 3A) of this value, and for scenario **c**, the relative infectivity just prior to the rash is 20% of the infectivity during the first week of the rash, and during the prodromal period is 20% of this value (these two parameters are the same as in Figure 3A).

Transmission prior to the rash makes epidemic control more difficult. In Figure 8, we show an expanding smallpox epidemic assuming differing levels of infectivity prior to the rash (adding increased infectivity prior to the rash, keeping constant the infectivity after the rash). We assume all parameters are the same as in Figure 3A (and that the ring vaccination capacity is 40 per day). Infectivity prior to the rash is modeled as the relative infectivity during the short (1 day) period of oropharyngeal lesions just prior to the rash (compared to the infectivity during the first week of the rash), and as the relative infectivity during the prodromal period (relative to the period just prior to the rash). We consider three scenarios: **a **relative infectivity during entire period is one (i.e., infectivity during the prodromal period and just prior to the rash is the same as during the first week of the rash), **b **the relative infectivity just prior to the rash is the same as during the first week of the rash, but during the prodromal period is 4% (as in Figure 3A) of this value, and **c **the relative infectivity just prior to the rash is 20% of the infectivity during the first week of the rash, and during the prodromal period is 20% of this value. The figure shows that increased infectivity just prior to the rash leads to a larger epidemic (comparing **b **and **c**); in case **b **(high infectivity just prior to onset of rash), loss of containment occurs 36% of the time (but in none of the 100 realizations shown in case **c **(low infectivity prior to rash)). Scenario **a **(full infectivity during entire the prodromal period) showed loss of control in every realization. Increasing the ring vaccination capacity from 40 per day to 80 per day (results not shown) led to containment in all of the realizations with high infectivity just prior to the rash and low infectivity during the prodromal period (case **b**), but made no difference if the infectivity was as high during the prodromal period as during the rash (case **a**). While intuitively adding additional infectiousness must increase the number of secondary cases and make control more difficult, these results do illustrate that even a small amount of increased infectiousness prior to the rash (when diagnosis is more difficult) may substantially increase the difficulty of smallpox control.

#### Other factors

**Figure 9:**
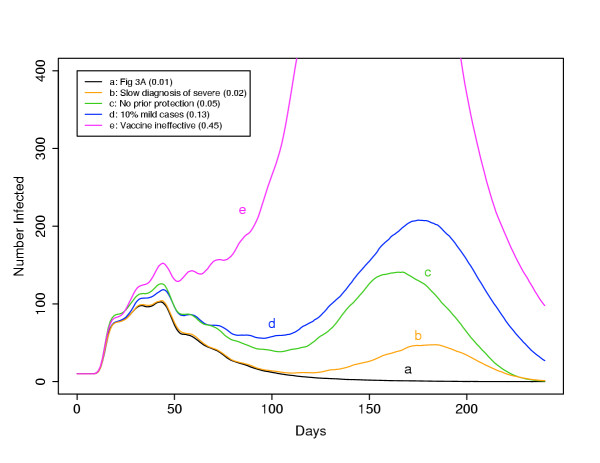
Additional scenarios, assuming 40 ring vaccinations or contact tracings possible per day, and that contacts of contacts are traced; all parameters are identical to those in Figure 3A unless otherwise indicated. The figure shows the average of 100 replications of five scenarios (Case **a **repeats the result from Figure 3A for reference); the numbers in parentheses in the legend are the corresponding fraction of the 100 scenarios for which decontainment occurred. For case **b**, we assumed that flat and hemorrhagic smallpox cases took four times as long on average to diagnose as ordinary cases; for case **c**., we assumed that no one in the population had prior protection (as opposed to 25% for Figure 3A); for case **d**, we assumed that an additional 10% of individuals (13% instead of 3%) would develop mild smallpox (with 75% developing ordinary smallpox instead of 85% as in Figure 3A); and for case **e**, we assumed that the vaccine is completely ineffective and provides no protection against infection.

Finally, in Figure 9, we present scenarios in which each of four other parameters are modified from the baseline values of Figure 3A, assuming 40 contact tracings (ring vaccinations) are possible per day (line **a **in the figure). Specifically, we assume that severe smallpox (hemorrhagic and flat) on average takes four times longer to diagnose and isolate than ordinary smallpox (case **b**), that no one in the population has prior vaccination protection (from before the discontinuation of routine vaccination, case **c**), that 10% more smallpox is too mild to diagnose (but still contagious, case **d**) compared to baseline, and finally that the vaccine is completely ineffective (case **e**). Each of these scenarios will be discussed further below.

Scenario **b **was motivated by the possibility that individuals with severe forms of smallpox may be more difficult to diagnose, and thus remain infectious in the community longer (despite the much greater degree of illness of such patients), or that such patients may be more infectious. In this particular case, quadrupling the mean diagnosis time led to one additional replication out of 100 in which containment was not achieved (2/100, compared to the baseline of 1/100). However, we assumed that community awareness of smallpox leads to the same relative rate of increased diagnosis among severe cases as for ordinary cases, and that the most severe forms are relatively rare. In addition to the scenario shown in the figure, we also replicated the same 1000 "calibrated" simulations, assuming that in each case 40 contact tracings per day are possible and that the diagnosis time for severe cases was four times that of ordinary cases. Finally, we repeated each "calibrated" scenario 100 times assuming long diagnosis times for severe cases, and not making this assumption, and found that the difference in the decontainment fraction was not large (results not shown).

Scenario **c **illustrates that vaccination prior to the discontinuation of routine vaccination does play a role in smallpox control by ring vaccination; there were more decontainment scenarios (5/100) when no prior protection exists in the population. The results suggest that prior vaccination aids in the control of smallpox, but that it is not strictly necessary for control (in this scenario, 95% of the replications exhibited containment). In Figure 3A, we assumed 25% of individuals had protection due to vaccination prior to the discontinuation of routine vaccination; in scenario **c **of Figure 9, we assumed this fraction was zero.

Scenario **d **demonstrates that if 10% more smallpox infections (in absolute terms, i.e. 13% compared to 3% in Figure 3A) lead to mild cases among individuals with no prior protection, the epidemic is more difficult to contain (13/100 replications showed loss of containment).

Finally, scenario **e **demonstrates that containment is still possible even when the vaccine is completely ineffective in everyone – because of case isolation and isolation of contacts (and of contacts of contacts). Here, with 40 contact tracings possible per day, 55% of the replications nevertheless exhibited containment even with a vaccine which offered no protection whatever. With 90 contact tracings possible per day, all replications exhibited containment even assuming no vaccine protection.

#### Effect of mass vaccination

**Figure 10:**
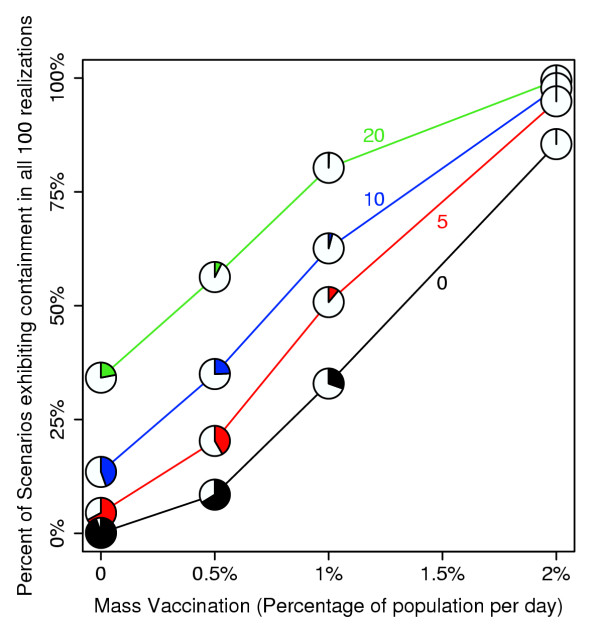
**Mass and ring vaccination **together. Low-level mass vaccination programs are improved substantially by the addition of ring vaccination. The shaded pie segments represent the fraction of 1000 scenarios for which containment (as defined in the text) was never realized; the vertical position of the pie chart represents the fraction of the 1000 "calibrated" scenarios for which containment was always achieved. As the fraction of the population mass vaccinated increases or the ring vaccination capacity increases, the probability of containment increases.

Although less efficient than ring vaccination in the sense that more vaccinations must be delivered to eliminate infection, comprehensive mass vaccination following the introduction of smallpox is sufficient to eliminate the infection. In Figure 10, we show the probability of achieving containment (defined to be fewer than 500 total cases resulting from 10 index cases) for different levels of ring vaccination (0, 5, 10, and 20 vaccinations per day) and mass vaccination (0, 0.5%, 1%, and 2%; compare with the 10%-20% per day many jurisdictions in the United States are planning to vaccinate). Specifically, for each level of ring vaccination and mass vaccination, we used the same 1000 parameter sets used in Figure 5, and performed 100 simulated epidemics for each parameter set. On the vertical axis, we plot the fraction of the 1000 scenarios for which each of the 100 simulated epidemics was contained. We further computed the fraction of scenarios for which none of the 100 simulated epidemics was contained; this is indicated by the colored segment in the small pie chart at each symbol. When the mass vaccination rate was 2% per day, the mean number of deaths (averaging over all scenarios and all simulations within each scenario) was 47.7, 33.7, 26.4, and 20.1 for a ring vaccination level of 0, 5, 10, and 20 per day (respectively) out of a population of 10000. Moreover, when we increased the mass vaccination level to 3%, an average of 28.9 deaths occurred when no ring vaccination was used, but this fell to 22.3 deaths when only 5 ring vaccinations per day were available (again assuming a population of 10000, and 10 index cases). With a mass vaccination level of 5% per day, an average of 18.8 deaths occurred without ring vaccination, and 15.8 deaths occurred when only 5 ring vaccinations per day were possible. (At a mass vaccination rate of 3% per day, containment as defined above was achieved in all 100 replications for 95% of the scenarios even without ring vaccination; at a mass vaccination rate of 5% per day, containment was achieved in all replications for all scenarios.) These results show that over a wide range of simulated epidemics, even seemingly small levels of ring vaccination (coupled with follow-up) may have a substantial effect in preventing epidemic spread and reducing deaths from smallpox, even during a mass vaccination campaign. Note that many jurisdictions in the United States are planning mass vaccination campaigns which could reach 10%-20% of the population per day, far greater than the mass vaccination levels examined here; it is interesting to note that mass vaccination campaigns may be effective in preventing a widespread epidemic even at much lower levels than are being planned for. Where feasible, such rapid mass vaccination rapidly eliminates smallpox transmission in our model; vaccination of contacts is still beneficial, since we are assuming that earlier vaccination yields a greater probability of preventing or ameliorating infection (results not shown).

## Discussion

We constructed a simple network model of smallpox transmission, and addressed the question of what circumstances contribute to the success of a ring vaccination campaign designed to control smallpox. Our analysis focused on the use of contact tracing/ring vaccination to prevent a widespread epidemic following a deliberate release.

We conducted a sensitivity analysis based on particular, but reasonable, ranges for the unknown parameters. Our results are consistent with prior vaccination models in identifying prior vaccination and ring vaccination capacity as significant factors in determining the spread of smallpox. Unsurprisingly, we also find that household size and ring vaccination speed are particularly important parameters; these results are intuitively plausible. The contact finding probability did not appear important in this analysis only because a narrow range of values was chosen.

We illustrated smallpox control by presenting scenarios based on control of moderately severe smallpox epidemics. We find that swift, aggressive contact tracing and ring vaccination is is usually sufficient to bring the infection under control. Provided that there is sufficient capacity, vaccination of contacts of contacts is beneficial, and results in fewer infected individuals and more rapid elimination of infection; investigating contacts of contacts allows the chain of transmission to be outrun to some extent. When ring vaccination capacity is small, diversion of crucial resources away from contacts is harmful; contacts of contacts should only be traced and vaccinated provided that no resources are diverted away from contacts of cases. The increased surveillance (or isolation) of contacts, together with improved rates of diagnosis due to community awareness, play important roles in smallpox control; we note that in some cases, lowered diagnosis rates among severe cases contributed to a small extent to loss of epidemic control, and suggest that any public awareness campaign include information to help the public be more aware of the full spectrum of the clinical features of smallpox.

One limitation of our analysis is that we chose not to explicitly incorporate the specific epidemiology of health care workers (or mortuary workers), who are likely to be exposed to infected individuals during any smallpox epidemic (e.g. [17,22]), and who may then infect further members of the community [22] (as was also seen in the recent outbreak of SARS, e.g. [48]). Transmission to health care workers may be considered to amplify the initial attack or to be simply accounted among the exposures we considered (and thus be approximated by the behavior of our model), since health care workers and their household contacts are in all likelihood traceable contacts, and ring vaccination/contact tracing would identify and halt these chains of transmission as in our model. The disruption of smallpox control and patient care that may occur is not accounted for in our analysis, however, causing our model in this sense to err on the side of optimism. The appropriateness of pre-event vaccination of health care workers or other first responders has been addressed by other analyses [12,49], and is beyond the scope of our model.

While we analyzed the effect of contact tracing, case and contact isolation, and ring vaccination (together with mass vaccination), in a real smallpox epidemic, in practice, control efforts are unlikely to be limited strictly to vaccinating contacts (and health care workers, as likely contacts) and isolating cases. Indeed, making vaccine available to individuals who believe they live near cases or to others on a voluntary basis occurred in smallpox control efforts in the past [22]. Vaccination of such individuals can only harm the disease control effort if it hinders or delays the diagnosis of cases or the investigation and vaccination of contacts; our results show that even relatively low levels of vaccination of the general population may have a beneficial effect in preventing the epidemic from escaping control.

More serious is the possibility that individuals who should be vaccinated or isolated would be missed; this could occur either because individuals or institutions did not cooperate with the disease control effort, or because the individuals simply could not be found. Our analysis suggests that ring vaccination need not be perfect to successfully contain the epidemic, and yet, under conditions where there is a high rate of infection among contacts, or a relatively high rate of casual transmission, high rates of contact finding (in excess of 90%), together with increased surveillance and contact isolation, are needed to contain the epidemic.

Finally, the vaccination of individuals at low risk of contracting smallpox will cause harm due to adverse events of the vaccine; in our model, the assumed death rate due to vaccination was small compared to the probability of death from smallpox, and played essentially no role in the analysis. In practice, individuals suspected to be at high-risk for vaccine complications, but at relatively low risk for contracting smallpox, might simply be isolated or closely monitored even during an outbreak; while the presence of individuals in the population at higher risk for vaccine complications would increase the death rate during an outbreak, such individuals are unlikely to impair the containment of the epidemic (the primary focus of this analysis).

Our results support ring vaccination against epidemics of smallpox (even assuming high rates of transmission to close contacts), but do note that stochastically, for severe (rapidly transmissible) smallpox, scenarios of loss of control are seen, with resulting widespread epidemics. In scenarios in which the transmission potential of smallpox is smaller, such loss-of-control scenarios occur less frequently (results not shown). Mass vaccination campaigns, when conducted quickly and with very high coverage, do not result in loss of control in our model. Nevertheless, fewer deaths due to smallpox result when ring vaccination is conducted along with mass vaccination.

## Conclusion

Simulated smallpox epidemics with ring vaccination suggest that aggressive, fast ring vaccination can control epidemics of smallpox. To do so, however, smallpox must be identified quickly and contacts vaccinated promptly. We also identify public awareness of smallpox – leading to prompt identification of cases – as a major factor in smallpox control; in some simulations, it may play a role as significant as ring vaccination itself [15]. However, we also found that uncertainty in (1) transmission from mild cases, (2) the household size, and (3) casual transmission contributed to the overall uncertainty in the epidemic size. Other parameters to which the number of infections were highly sensitive were the prior vaccination fraction, parameters related to infectiousness, and parameters related to transmission prior to the rash.

Because our model combines network structure with response logistics, our results support and complement the results of other investigators. Our results support the notion that prior vaccine protection may play an important role in slowing the epidemic [11], despite the possibility that some vaccinated individuals may develop mild cases which are harder to identify, but which nevertheless transmit disease. Likewise, our results provide support for the view that ring vaccination should play a central part in smallpox control. If initiated, ring vaccination should be conducted without delays in vaccination, should include contacts of contacts (whenever there is sufficient capacity to cover all contacts of cases), and should be accompanied by a vigorous campaign of public awareness *which can facilitate more rapid identification and isolation of cases. *We assumed that ring vaccination could be fast (little delay between identification of a case and vaccination of the contacts), effective (nearly all household contacts can be found, and most of workplace and social contacts), and available (there is sufficient capacity). To be effective, ring vaccination planning must yield a system capable of meeting these benchmarks; we should not only be able to assess the number of contact vaccinations that will be possible per day, but should have a plan in place to (1) identify contacts by working with individuals, employers, schools, community representatives, and authorities or businesses who may have access to information facilitating contact tracing, (2) rapidly investigate and vaccinate such individuals, perhaps using field teams managed by central dispatch. It is important to realize that for high-risk, transient, or unstably housed populations where reliable contact tracing is impossible, the conclusions of the model we present cannot be applied. It is important to note that while our model suggests that ring vaccination together with contact tracing and isolation is likely to be successful, we found that for some scenarios (where smallpox was more transmissible, or was relatively more transmissible before the rash), epidemic containment required not only ring vaccination, but increased public awareness, the isolation of contacts, and tracing of contacts of contacts. For scenarios in which the smallpox was less transmissible, epidemic containment was possible at lower contact finding probabilities. Thus, while our simulations suggest that contact tracing/ring vaccination need not be perfect to succeed, because of uncertainties in our knowledge of the behavior of bioterrorist smallpox, it is impossible to know in advance how good it will have to be. Thus, that high contact finding rates, mass public awareness leading to early identification of cases, isolation of contacts, and investigation of contacts of contacts should all be conducted with maximum effectiveness to reduce the probability of a widespread epidemic.

While the possibility of smallpox uncontrollable by ring vaccination has made mass vaccination preparations wise, and while mass vaccination may be unavoidable in the event of a deliberate release of smallpox, we believe that ring vaccination is essential in any case. This is not only because individuals recently exposed to smallpox may be protected if they are vaccinated promptly, but because each contact identified potentially lies in the immediate future of the transmission chain. From the standpoint of epidemic control, it is far more valuable to vaccinate individuals next in the transmission chain than to vaccinate other persons. Our results support the idea that ring vaccination/case isolation may in many, if not most cases, eliminate smallpox even without mass vaccination, but also support planning for mass vaccination (so that the vastly more costly and difficult policy of mass vaccination will be available in the event of an explosive epidemic). When faced with the unknown, multiple redundant preparations are appropriate; case investigation/isolation may control smallpox even if the vaccine does not work at all, but mass vaccination is useful in the event of an explosive epidemic for which case tracking becomes impossible.

## Competing interests

None declared.

## Authors' contributions

TP, KH, SF, TA, RR, and DP performed the literature review (and parameter evaluation), TP developed and implemented the model and simulation, TP performed the analysis of the simulation model and drafted the manuscript, DP performed analysis of contact tracing data, and KH conceived of the study. All authors contributed to, read and approved the final manuscript.

## Pre-publication history

The pre-publication history for this paper can be accessed here:



## Supplementary Material

Additional file 1 For consistency, all references are included in the bibliography of the main text. Click here for file

Additional file 2 For consistency, all references are included in the bibliography of the main text. Click here for file
