# Supplementary material for: Logistics of community smallpox control through contact tracing and ring vaccination: a stochastic network model
Source: BMC Public Health. 2004 Aug 6;4:34. doi: 10.1186/1471-2458-4-34 (PMC520756; doi:10.1186/1471-2458-4-34)
Supplement: Additional file 1 — For consistency, all references are included in the bibliography of the main text. [file 1471-2458-4-34-S1.pdf]

Appendix 1 for "Logistics of community smallpox control through contact tracing and ring vaccination: a stochastic network model"

Travis C. Porco[1,2,3], Karen Holbrook[1], Susan E. Fernyak[1], Diane Portnoy[1], Randy Reiter[1], and Tomás J. Aragón*[2,1]

1 San Francisco Department of Public Health, Community Health and Epidemiology Section, Epidemiology and Effectiveness Research Unit, 101 Grove Street Suite 204, San Francisco, California 94102 USA
2 Center for Infectious Disease Preparedness, School of Public Health, University of California, Berkeley, USA
3 Present address: Surveillance and Epidemiology Section, Tuberculosis Control Branch, Division of Communicable Disease Control, California Department of Health Services, Berkeley, California, USA

* To whom correspondence should be directed: Tom'as J. Arag'on, M.D., Dr.P.H., San Francisco Department of Public Health, Community Health and Epidemiology Section, Epidemiology and Effectiveness Research Unit, 101 Grove Street Suite 204, San Francisco CA 94102 USA; email: `aragon@ucbcidp.org`; phone: (415) 554-2724; fax: (415) 554-2854.

In this Appendix, we give further details on the smallpox severity classification; see main text for references.

Early hemorrhagic smallpox [26] was rapidly and uniformly fatal, but occurred in only 0.7% of Rao's unvaccinated subjects. This form of smallpox (which seems to be equivalent to *purpura variolosa* or *fulminating smallpox* [17]) was often attributed to a deficiency in the immune response, rather than to unusually virulent strains [31, 26], and occurred most commonly among pregnant women [26]. Among subjects vaccinated, the proportion of early hemorrhagic cases was 1.4%, and if a person who had been vaccinated developed this form of smallpox, there was believed to be no amelioration or modification of the clinical course. The epidemiological significance of this form lies in its rapid fatality and in the diagnostic difficulties it engendered; for example, in the Stockholm outbreak of 1963 [22], one person who died of what was later diagnosed as *purpura variolosa* infected two household contacts and one hospital worker. In patients with this form of smallpox, death occurs within a few days; we assume a survival of 1–6 days.

We aggregate two other severe forms of smallpox which were characterized by very high case fatality rates and survival times of approximately 8–12 days. *Late hemorrhagic* smallpox [26] occurred in 1.7% of Rao's unvaccinated subjects and in 2.0% of Rao's vaccinated subjects; this form of smallpox was characterized by hemorrhages into the bases of the lesions. *Flat* smallpox occurred in 6.7% of unvaccinated subjects and in 1.3% of vaccinated subjects; this form was marked by soft, flat, slowly maturing lesions unlike the classical "focal" rash. The case fatality rate was 96% among unvaccinated subjects and

67% among vaccinated subjects [26]. Flat smallpox appears to have been similar to Dixon's *malignant* categories; however, in Dixon's case series in Tripolitania, malignant forms appeared to be more frequent and less fatal [50, 17] than in Rao's case series from Madras. The epidemiological significance of these two forms of smallpox again lies with their high case fatality rates and potential diagnostic difficulties; one case of hemorrhagic smallpox yielded 38 secondary cases during the Yugoslavian outbreak of 1972 [26]. Unlike for early hemorrhagic smallpox, prior vaccination did seem to lower the fatality rate.

Ordinary type smallpox accounted for 89% of Rao's unvaccinated cases, and displayed an overall case fatality rate of 30.2%; such cases were subdivided according to the density of lesions as confluent, semiconfluent, and discrete [26], with the case fatality rate higher when more lesions were present. Among vaccinated individuals, 70.0% of Rao's subjects were considered to have ordinary type smallpox, and experienced a case fatality rate of 3.2%.

Modified type smallpox accounted for only 2.1% of the subjects classified by Rao as unvaccinated, and in 25% of Rao's vaccinated subjects [26]. In modified type smallpox, the pre-eruptive phase was often severe and of the usual duration, but the skin lesions were less uniform, more superficial, and evolved more rapidly [26, 17]. The case fatality rate was 0%. The epidemiological significance of this form was in its lowered fatality rate, and in the fact that such individuals were believed to transmit less disease [32].

Finally, some subjects experienced very mild cases of smallpox, exhibiting very few lesions, "abortive" infections [17], or *variola sine eruptione* (the usual smallpox initial phase, but no rash) [17, 26]. Although very mild cases were more common among vaccinated individuals,

such cases did occur in unvaccinated individuals from time to time. The epidemiological significance of such cases is that while they may be less infectious in the sense of shedding less virus, such individuals may not seek diagnosis and their normal activities may be unimpeded, leading to more transmission. In the Stockholm outbreak [22], it was thought possible that two health care workers with mild cases may have resulted in a single transmission to an infant, while the Yugoslav outbreak of 1972 [26] was initiated from a traveler with a mild case. We assume that between 0 and 5% of unvaccinated subjects would develop mild smallpox, and that between 1 to 3 times this proportion of vaccinated individuals would develop mild forms. Given that an individual did not develop mild smallpox, we utilize the proportions given by Rao [26] to determine the proportion who develop early hemorrhagic, late hemorrhagic or flat, ordinary, and modified type smallpox. Some evidence suggests that smallpox may have manifested different spectra of severity in different places and times, though the difficulties in comparing assessments of different observers in different places and times makes such conclusions tentative. Epidemics of *variola minor* exhibited very low case fatality rates [51]; at the other extreme lay, for instance, the outbreak of smallpox in Windsor, Ontario in 1924, which displayed an overall unvaccinated case fatality rate of 71%, and 11 (all fatal cases) out of 37 cases exhibited rashes considered unusual by the investigators [52]. Bioweapons developers would be expected to attempt to develop or utilize strains of greater virulence than the usual *variola major*; such considerations cannot be ignored simply because of the lack of reliable information in the open literature.
