# Supplementary material for: Logistics of community smallpox control through contact tracing and ring vaccination: a stochastic network model
Source: BMC Public Health. 2004 Aug 6;4:34. doi: 10.1186/1471-2458-4-34 (PMC520756; doi:10.1186/1471-2458-4-34)
Supplement: Additional file 2 — For consistency, all references are included in the bibliography of the main text. [file 1471-2458-4-34-S2.pdf]

Appendix 2 for "Logistics of community smallpox control through contact tracing and ring vaccination: a stochastic network model"

Travis C. Porco[1,2,3], Karen Holbrook[1], Susan E. Fernyak[1], Diane Portnoy[1], Randy Reiter[1], and Tomás J. Aragón[*2,1]

1 San Francisco Department of Public Health, Community Health and Epidemiology Section, Epidemiology and Effectiveness Research Unit, 101 Grove Street Suite 204, San Francisco, California 94102 USA
2 Center for Infectious Disease Preparedness, School of Public Health, University of California, Berkeley, USA
3 Present address: Surveillance and Epidemiology Section, Tuberculosis Control Branch, Division of Communicable Disease Control, California Department of Health Services, Berkeley, California, USA

* To whom correspondence should be directed: Tom'as J. Arag'on, M.D., Dr.P.H., San Francisco Department of Public Health, Community Health and Epidemiology Section, Epidemiology and Effectiveness Research Unit, 101 Grove Street Suite 204, San Francisco CA 94102 USA; email: `aragon@ucbcidp.org`; phone: (415) 554-2724; fax: (415) 554-2854.

Appendix 2

In this Appendix, we provide a full specification of the continuous-time, discrete-event system which constitutes our model (using the terminology of Glasserman and Yao [53]); see main text for references. Thus, we specify five components: (1) the state space of the model, (2) the set of possible events, (3) the set of active events for each possible state, (4) the transition function which specifies how the state of the system changes when an event occurs, and finally (5) the parameter values. Each active event has an event time associated with it; the simulation proceeds by choosing the active event with the smallest event time. The state of the system is then transformed according to the chosen event [53], and the simulation time t is updated to the time of the event which occurred. This process yields a sequence of event occurrence times $t^0, t^1, \ldots, t^k, \ldots$ and a corresponding sequence of state values at these times $\mathbf{X}^0, \mathbf{X}^1, \ldots, \mathbf{X}^k, \ldots$, for each event time index $k = 0, 1, \ldots$. Thus, $\mathbf{X}^0$ is the initial condition of the system at time t^0; the first event occurs at time t^1 and causes the state of the system to change from $\mathbf{X}^0$ to $\mathbf{X}^1$, and so forth. When such a state change takes place, new events may become active (and their event times must be determined); other events may no longer be active (and they must be removed from the event list). We first specify the state $\mathbf{X}$ of the system, and then each of the possible events (and the transitions that result).

State specification

States of individuals

We first specify the possible states of the individuals in the model. Individuals are indexed by j, $j = 1, 2, \ldots, N$, where N is the number of individuals in the population. The state of individual j is represented by the j-th component of one of the vectors given in Table 5. Uninfected individuals have a stage G_j of zero; otherwise, the stages are indicated in Figure 1. We define Z_j to be 1 if person j has been very recently vaccinated (within a few days or a week, discussed further in this Appendix), and zero otherwise.

It is convenient to define I_j as an indicator of whether person j is infectious, i.e. I_j is 1 if G_j is 3, 4, 5, 6, or 7, and 0 otherwise; also, let Y_j indicate whether individual j is infected, i.e. Y_j is 0 if G_j is 0 or 9, and is 1 otherwise. Finally, it is convenient to define Q_j as an indicator of whether person j is in the symptomatic pre-eruptive phase, i.e. whether G_j is 4 or 5.

Smallpox severity. For uninfected individuals, the pox severity S_j is defined to be zero; the severity classes 1–5 correspond to mild, modified, ordinary, flat, and hemorrhagic.

Smallpox stage. For ordinary-type smallpox, we assume the following stages. We assume that for individuals j who have never been infected, the stage $G_j = 0$. The stages are indicated in Figure 1 and summarized in Table 6. For hemorrhagic smallpox, the mortality rate is essentially 100%, and we assume that the individual will not progress through all the stages.

Recent vaccination indicator. When individual j is just vaccinated, we set $Z_j = 1$; otherwise $Z_j = 0$. The value $Z_j = 1$ indicates that an individual has just been vaccinated, but has not had time to develop full vaccine protection nor to develop fatal complications.

Visit generation. When an individual has never been seen by a doctor to receive a diagnosis, or has never been visited as a result of a case investigation, $V_j = 3$. If individual j is diagnosed as a case of smallpox, $V_j = 0$. If individual j is investigated because they are a contact of a smallpox case, but is not diagnosed as a case as a result of the visit, then $V_j = 1$ (and the person is a non-case contact of a case). If individual j is investigated because they are a contact of a non-case contact of a case, but is not diagnosed as a result, then $V_j = 2$.

Immunity status. We assume that an individual is in one of five immunity classes: unexposed/unvaccinated, successfully vaccinated 20 or more years ago, successfully vaccinated between 3 and 19 years ago (we don't use this category in the results we present, but indicate it for completeness), successfully vaccinated prior to infection and within the last three years, successfully vaccinated after infection within the "window period", and naturally immune. These are used to determine the probability that an individual will be protected from infection, and the severity class of smallpox if an individual is infected.

Protection status. For each individual, the variable P_j indicates whether or not the person will become infected if exposed. For individuals with no immunity ($M_j = 0$), $P_j = 0$; for individuals with natural immunity (due to smallpox infection), $M_j = 4$ and $P_j = 1$. For other values of M_j, the protection status will be determined as explained below.

Alert status. If a person is visited as the result of an investigation (because they are a contact of a case or a non-case contact of a case), they will be diagnosed if they are symptomatic. If they are not symptomatic, we assume that (in addition to vaccination) they will be closely monitored so that they will be diagnosed more quickly should they develop symptoms. When individual j is alerted, $A_j = 1$; otherwise $A_j = 0$.

Global variables

Additionally, the following scalar variables are defined: K: the ring vaccination capacity per day; G: 1 if the community is aware that a smallpox epidemic is occurring, 0 otherwise; M: 1 if a mass vaccination campaign is occurring, 0 otherwise; and C, the cumulative number of diagnosed cases.

Full state space

The full state space $\mathbf{X}$ of the system is specified by the collection

$$\mathbf{X} = \{S_j, G_j, U_j, V_j, A_j, P_j, M_j, D_j, Z_j, \theta_j, K, G, M, C\},$$

where S_j denotes the collection of severity values for each individual j, etc.

Initial conditions

The model is initialized with N individuals. We assume that all individuals are alive ($D_j = 0$ for all j), uninfected ($S_j = 0$ and $G_j = 0$ for all j), undiagnosed ($U_j = 1$ for all j), unalerted ($A_j = 0$ for all j), unvisited ($V_j = 3$ for all j), and not recently vaccinated

($Z_j = 0$ for all j). We assume that a fraction f of individuals have old vaccine protection ($M_j = 2$); for such individuals, $P_j = 1$ with probability α_2 and $P_j = 0$ otherwise. All other individuals have no old vaccine protection, so that $M_j = 0$ and $P_j = 0$. In general, we define α_i to be the probability that an individual in immune class i will be protected from infection (see Table 5 for a summarization of the immunity classes), and refer to this as the vaccine success rate (Table 8).

The connection matrix $\mathbf{C}$ is determined by assigning individuals to households of size H, and by randomly assigning a fraction w of individuals to workplace/social groups of size W. When individual j is a household contact of individual i, $\mathbf{C}_{ji} = 1$; when individual j is a workplace contact of individual i (but not a household contact of individual i), $\mathbf{C}_{ji} = 2$; otherwise $\mathbf{C}_{ji} = 0$. We further determine the elements of the matrix $\mathbf{F}$, where $\mathbf{F}_{ji}$ which indicates whether or not j can be found by tracing from i; ν_c is the probability of tracing a contact (where $c = 0$ indicates no contact, and $\nu_0 = 0$; ν_1 is the probability of finding a household contact, and ν_2 is the probability of finding a workplace/social contact). Then $\mathbf{F}_{ji}$ is 1 with probability $\nu_{\mathbf{C}_{ji}}$ and 0 otherwise.

The epidemic is initiated by selecting A individuals at random and scheduling infection events (see below) for them at time $t^0 = 0$, the beginning of the simulation.

Events

The set of possible events is listed in Table 7. The event times and transitions associated with each of these will be discussed in turn. Any state variables (components of $\mathbf{X}$) whose

values are not otherwise specified for any particular event are assumed to remain unchanged from $\mathbf{X}^k$.

Progression events. For each individual j in the population who is infected ($Y_j = 1$), a progression event $\mathcal{P}_j$ is active. The time that must elapse before the next progression event occurs is determined by sampling from a uniform distribution with a specified minimum and maximum (depending on the stage and severity, as indicated below), except for the transition from stage 2 to 3 (corresponding to the beginning of symptoms). These are not exponentially distributed waiting times and the progression process is thus not Markovian; the distributions we used were chosen as a simple, flexible, and computationally efficient way to ensure that a minimum residence time is spent in each state.

For all five severity classes, the lower bound of the duration of the first stage (indexed by $g = 1$, the window period for which vaccination may provide protection) is chosen from the range 1.5–2.5 days, and the upper bound is computed by adding a number chosen from the range 0.5–1.5 days to it. The lower bound of the duration of the second stage (indexed by $g = 2$, the window period for which vaccination will not prevent disease but may provide amelioration) is also chosen from the range 1.5–2.5 days, and the upper bound is computed by adding a number chosen from the range 0.5–1.5 days to it. Thus, the first window period may last from 2–4 days, and the remaining stage may also last from 2–4 days; Dixon (1962) writes that successful vaccination up to one week after exposure may prevent or modify disease in up to 50% of those vaccinated.

To determine the incubation period, i.e. how much time must elapse before the transition

from stage 2 to stage 3 will occur, we first determine how long the person has been infected ($t^k - \theta_j^k$), and subtract this from the incubation period for person j. The incubation period for person j is determined by sampling from a beta-distributed random variable with parameters $b_1 = 3.88$ and $b_2 = 6.08$, scaled to the interval (7, 19) days. This distribution parameterizes the incubation period distribution derived from imported cases in Europe with a known exposure time [26].

For all five severity classes, we select the lower bound of the duration of stage 4 (symptomatic, pre-eruptive) from the range 1–2 days; we select the upper bound by adding a number chosen from the range 1–2 days to the lower bound. This yields a range of 2–4 days for the symptomatic, pre-infective period. For stage 5 (infectious, but pre-eruptive), we choose the lower bound from the range 0–1 and the upper bound by adding a number chosen from 0–1 to it (for a total range of 0–2 days). Clinical observations support the idea that the period of pre-eruptive constitutional symptoms preceding the focal rash may be 2–4 days, and that the enanthem (oropharyngeal lesions, which are believed to facilitate airborne or droplet transmission) may appear 24 hours before the rash itself [26]. The remaining model stages represent the period of the smallpox rash; they approximate maximum infectivity, maximum death risk, and convalescence, respectively. For flat, ordinary, and "mild" smallpox, for each of the three stages, the lower bound was chosen from 5.5–6.5 days, and the upper bound was chosen by adding a number from the range 1.5–2.5 days to the lower bound. For modified type smallpox, we accelerate the progression; the lower bound is chosen from 4.5–5.5 days and the upper bound by adding

1.5–2.5 days to the lower bound.

When a progression event occurs for person j, then $S_j^k = S_j^{k-1} + 1$; if $S_j^k = 9$ then, the person has recovered, and so $M_j^k = 4$, $P_j^k = 1$, and $S_j = 0$. When an individual recovers, the death events $\mathcal{D}_j$ and diagnosis events $\mathcal{V}_j^0$ associated with that individual are no longer active. When an individual progresses into stage 4–8, diagnosis events may become active. For individuals with flat, ordinary, modified, or mild smallpox, when a transition to stage 7 occurs (to stage 5 for hemorrhagic cases), a death event becomes active for individual j. The severity-specific case fatality rate is denoted δ_s (for severity class s), so with probability δ_s the person will be a smallpox fatality. If the person is to be smallpox fatality, then the time is selected from the progression time distribution (and the progression event is given event time ∞); if the person is not to be a smallpox fatality, then the death event is given time ∞. It is important to note that this model is intended to simulate smallpox control in developed countries, and we always assume that an individual will be rapidly diagnosed and that once diagnosed, the individual will be isolated (in particular, no sickbed transmission will occur); for this reason, the actual duration of symptoms and the time of death have limited significance. If sufficient isolation capacity is unavailable and substantial transmission to health care workers or other patients occurs, then our model would not apply without revision.

Smallpox death events. Whenever a death event happens for person j at time t^k, all other events for person j become inactive, and $D_j^k = 1$, $S_j^k = 0$, and $G_j^k = 0$. Death is assumed to occur in stage 7 (if it occurs for non early-hemorrhagic cases) because most

deaths would occur on days 10–16 [26]. The parameters are chosen so that hemorrhagic smallpox is always fatal. The case fatality rate for flat-type smallpox is chosen from the range 0.5–0.8; the case fatality rate for ordinary-type smallpox is chosen from the range 0.1–0.3, and the case fatality rate for modified-type smallpox is chosen from the range 0–0.03. Mild smallpox is assumed to be never fatal.

Infection events. Infection events are active whenever there is at least one individual who is infectious ($I_i = 1$) and undiagnosed ($U_i = 1$) in the community, and at least one unprotected individual j ($P_j = 0$). The waiting time to infection for unprotected individual j is assumed to be exponential with rate (i.e., hazard) computed as follows. For every uninfected, unprotected person j in the population, each infective in the population contributes a baseline hazard for casually (randomly) infecting individual j; we denote this hazard by β. The actual hazard rate from every infective is found by multiplying β by the relative infectivity b_{sg} for the severity s and stage g for the infective. We denote the hazard of infection for household contacts by λ (initial numerical values chosen from the range 0.1–4). The relative hazard for different types of close contact is denoted h_c, for $c = 0, 1, 2$; $h_0 = 0$ for no close contact, $h_1 = 1$ for household contacts, and $h_2 \leq 1$ for social/workplace contacts relative to household contacts (numerical range chosen from 0–1). The infection hazard for person j is then

$$(1 - P_j) \sum_{i=0}^{N} b_{S_i G_i} I_i U_i \Big(\lambda h_{\mathbf{C}_{ji}} + \frac{\beta}{N} \Big).$$

To model the relative infectivity, we assume that the highest infectiousness is seen in the first week of the rash for flat and ordinary type smallpox (stage $g = 6$); we use this as the

baseline (so the relative infectivity is by definition 1). For the next two stages, we assume that the relative infectivity is multiplied by a factor between 0 and 1. The short stage just prior to the rash ($g = 5$) has a relative infectivity in the range 0–1. To find the relative infectivity of stage $g = 4$, we multiply the relative infectivity of stage $g = 4$ by an additional factor chosen from the range 0–1. The relative infectivity in modified-type smallpox is computed for each stage by selecting a factor from the range 0–1, and multiplying it by the corresponding relative infectivity for ordinary smallpox. We compute the relative infectivity for mild smallpox in the same way, except that we use a factor chosen from the range 0–0.5. Hemorrhagic smallpox is assumed to have the same infectivity as ordinary and flat smallpox. These values are assigned so that the unvaccinated secondary attack rate for household contacts is 0.5–1.0; Dixon estimates "that the natural attack rate in a modern community, with good housing, reasonably early diagnosis, and removal to the hospital, would be about 50% of those exposed" [17]. Evidence suggests that transmission rarely occurs from casual contact, and yet that smallpox can be sustained even in small groups [54]. Dixon estimated the all-age chance of being infected due to casual contact to be approximately 10%, and from living in an invaded house to be 75% [17]. We chose the hazard β for infection from the range 0–5 per infective per day—a range designed to allow a very wide range of contributions due to casual infection.

When an infection event occurs for person j, all other infection events for j become inactive, S_j is calculated as a multinomial random sample from the severity distribution

σ_{M_j} for the individual's underlying immune status, and $G_j = 1$. A progression event becomes active (for progression from stage 1 to stage 2), and the time that must elapse before this progression to occur is a sample from a uniform distribution as discussed previously.

The values that were chosen for the severity distribution we used were calculated as follows. Because five probabilities must be chosen that must add up to 1, we chose to compute them from independently varying conditional probabilities. We first chose the probability of ordinary-type smallpox (severity class 2), then the probability of flat or late hemorrhagic given that a person does not have ordinary-type, and then the probability of early hemorrhagic given that they do not have flat, late hemorrhagic or ordinary type. Finally we compute the probability of modified type given that a person does not have one of the previous three. These four probabilities, for individuals with no immunity, are chosen, respectively, from the ranges 0.8–0.9, 0.5–0.6, 0.2–0.25, and 0.4–0.5. The upper bounds of this range yield 90% ordinary smallpox, 6% flat type, 1% early hemorrhagic, 1.5% modified, and 1.5% mild smallpox, which is similar to the data from C. R. Rao's Madras series [26]. For all protected classes, we used the ranges 0.6–0.75, 0.05–0.1, 0.1–0.444, and 0.8–0.9 unless otherwise indicated in the text; the upper bounds yield 75% ordinary type, 2.5% flat type, 1% early hemorrhagic, 19.35% modified, and the remaining cases mild.

Events to diagnose cases without tracing. When individuals are symptomatic, they may seek medical attention and receive a diagnosis. We assume that such individuals do not utilize ring vaccination capacity. For every undiagnosed ($U_j = 1$), symptomatic

($4 \leq G_j \leq 8$) individual, a diagnosis visit $\mathcal{V}_j^0$ is active. We denote by γ_{sg} the severity- and stage-specific diagnosis rates. We assume that when the community is aware of a smallpox outbreak, diagnosis will occur faster; we set $a_0 = 1$ and let a_1 denote the relative rate of diagnosis when there is community awareness (numerical range 1–2). We set $f_0 = 1$ and let f_1 denote the further increase in diagnosis rate for alerted individuals when compared to unvisited individuals (numerical range 1–2). Finally, because in some scenarios, we may wish to assume that the pre-eruptive diagnosis rate is zero unless a person is being specifically monitored, we also define ϕ as the rate of diagnosis of pre-eruptive smallpox in pre-eruptive individuals under surveillance; large values of this parameter correspond to essentially immediate removal of such individuals, and thus to their effective isolation. We choose parameters so that the diagnosis rate in eruptive individuals is always greater than for pre-eruptive individuals, however, in all cases. The time that must elapse for a diagnosis is assumed to be exponentially distributed with rate

$$U_j(\gamma_{S_j G_j} a_G f_{A_j} + Q_j s)$$

for individual j. If this time is greater than the residence time in the stage, the diagnosis event is not active. Surveillance of contacts is a sufficiently important process that alternative models may yield different numerical (though qualitatively similar) results; we omit these results for brevity.

We wish to take into account the possibility that individuals will seek diagnosis and care even during the prodromal period (where the fevers could reach 104 Fahrenheit). For the baseline scenario in Figure 3A, we assumed a diagnosis rate of 0.5 per day for ordinary

type smallpox in the prodromal period, and of 1.25 per day after the onset of the rash; we assumed a diagnosis rate of 0.3 per day for severe smallpox (since some cases of hemorrhagic smallpox displayed lower fever during the prodromal period), and 1.5 per day after the onset of rash (due to the greater severity of illness). However, the diagnosis rate during the prodromal period made little difference in the outcome of the model, as we show in the text.

When a diagnosis occurs, $U_j^k = 0$, $V_j^k = 0$, and $C^k = C^{k-1} + 1$; if $C^k \geq 1$, then $G^k = 1$ (we assume no delay between the first diagnosis and complete community awareness), and if $C^k \geq V_t$ (the number of cases has reached the mass vaccination threshold), then $M^k = 1$ (a mass vaccination campaign is initiated). Also, when a diagnosis occurs, all other diagnosis and investigational visit events for the individual become inactive.

For ordinary and modified smallpox in the eruptive period, we assume the mean time to diagnosis is between 0.5 and 2 days; for pre-eruptive stages, we assume the diagnosis rate is between 10% and 50% of the rate during the eruptive period. For flat or hemorrhagic smallpox, we assume the mean time to diagnosis (and isolation) is between 0.4 and 1.333 days (because of the greater severity of symptoms); mild cases are not diagnosed.

Investigational visit events. Investigational visit attempts are active for all individuals j which have never been visited, and who can be reached from contacts that have been visited (provided person j is not too far removed from the source case). We denote by τ the farthest remove from the case we are willing to trace: when we assume that only contacts of cases are traced, then $\tau = 1$; when we assume that in addition, contacts of contacts of

cases (contacts, who have not been diagnosed as cases) are traced, then $\tau = 2$. Once a contact is identified, we assume that δ_c is the rate of finding the individual (the reciprocal of the delay) for a contact of type c (household or workplace/social). The time until event $\mathcal{V}_j^1$ (an attempt to trace person j as a contact of a case) is assumed exponential with rate

$$1\{V_j \geq 2\} \sum_{i=0}^{N} 1\{V_i = 1\} F_{ji} \delta_{\mathbf{c}_{ji}} 1\{K > 0\};$$

the person must either have never been visited, or have only been visited as a contact of a contact ($V_j = 3$ or $V_j = 2$), there must be at least one case among the contacts of j such that j can be reachable from the case, and there must be sufficient contact tracing/ring vaccination capacity. Similarly, the time until event $\mathcal{V}_j^2$ occurs for person j (tracing person j as a contact of a contact) is assumed exponential with rate

$$1\{\tau = 2\} 1\{V_j = 3\} \sum_{i=0}^{N} 1\{V_i = 2\} F_{ji} \delta_{\mathbf{c}_{ji}} 1\{K > 0\}.$$

We assume the delay time is 1–2 days for a household contact, and 2–3 days for a workplace/social contact.

When an investigational visit occurs, either the person is symptomatic or not; if the person is symptomatic, they are diagnosed; if they are not symptomatic and have not been infected, the person is alerted ($A_i^k = 1$) and if the person has not been vaccinated (i.e. $M_j^k = 0$ or $M_j^k = 2$), then the person is vaccinated (see below) provided there is sufficient ring vaccination capacity at the time ($K^{k-1} > 0$); and all investigational visit events use ring vaccination capacity, so that $K^k = K^{k-1} - 1$.

As before, when a diagnosis occurs, $U_j^k = 0$, $V_j^k = 0$, and $C^k = C^{k-1} + 1$; if $C^k \geq 1$, then

$G^k = 1$ (we assume no delay between the first diagnosis and complete community awareness), and if $C^k \geq V_t$ (the number of cases has reached the mass vaccination threshold), then $M^k = 1$ (a mass vaccination campaign is initiated). Once again, when a diagnosis occurs, all other diagnosis and investigational visit events for the individual become inactive. The visit variable V_j records the degree of remove represented by the investigational visit: if $\mathcal{V}_j^a$ occurs for person j, then $V_j = a$ (for $a = 0, 1, 2$). In this model, a person is only visited once at each degree of remove; if a person is investigated as a contact of a case, they need not be visited again (since they are already under surveillance). Similarly, if a person is investigated as a contact of a contact, then they will not be visited again as a contact of a contact, though they may be visited if one of their direct contacts subsequently becomes a case. Because contact tracing is modeled itself as a branching process wherein each visit yields further contacts and then visits (stopping after a certain number of removes), an individual who is a contact of a case could be reached first as a contact of one of the other contacts of the case. If each person were only visited once, then their contacts would not then be traced even though the individual was in fact a smallpox case; such assumptions would yield artificial underestimate of the efficacy of ring vaccination (results not shown); use of the variable V_j as in this model avoids this problem.

Vaccination of individuals in a mass campaign. Mass vaccination events for every individual are active whenever a mass vaccination campaign is occurring ($M = 1$). These are scheduled by choosing a random ordering of the population, and scheduling K_m (the mass vaccination capacity per day) individuals to be vaccinated during the day. A fraction

q_r of individuals respond to the mass vaccination campaign, and of these a further fraction q_e are eligible (so that the total probability of vaccination is $q_r q_e$). Eligible individuals who responded are then vaccinated at an event time chosen uniformly throughout the day. We chose the response fraction q_r from the range 0.9–1, and the eligibility fraction from the range 0.9–1.

When a mass vaccination event occurs for individual i, then $Z_i^k = 1$.

When individual j is vaccinated (either by investigation (ring vaccination), or due to mass vaccination), then $Z_j^k = 1$ indicating very recent vaccination. When an individual is vaccinated, the individual will die with probability v, at time D_v from the vaccination time (we assume $v = 1.0 \times 10^{-6}$ [55]), and $D_v = 10$ days. Assuming that the individual will not die, the full vaccine protection is attained at time D_f (we assume, 7 days). Thus, two events become active whenever $Z_j^k = 1$: death from vaccine complications ($\mathcal{C}_j$), and the achievement of the fullest protection for the individual ($\mathcal{F}_j$). The event times associated with these are calculated as follows: let Υ_1^k be a Bernoulli random variable with parameter ζ indicating whether or not the vaccine takes [56]; if $\Upsilon_1^k = 0$, then the event times for $\mathcal{C}_j$ and $\mathcal{F}_j$ are both ∞, and all components of the state $\mathbf{X}$ (except Z_j) remain unchanged. Assuming that the vaccine takes in individual j, we let Υ_2^k be a Bernoulli trial with parameter v indicating whether or not the person will die of vaccine-related complications. If $\Upsilon_2^k = 1$, then the event time associated with $\mathcal{C}_j$ is $t + D_v$; otherwise, this event time is ∞. The event time associated with $\mathcal{F}_j$ is $t + D_f$ if $\Upsilon_2^k = 0$ and ∞ if $\Upsilon_2^k = 1$. When the vaccine has taken, the further state transitions which occur depend on whether or not the

individual is (1) uninfected, (2) infected, in stage 1, or (3) infected, in stage 2. If the individual is uninfected, then $M_j^k = 1$ indicating very recent vaccination. We determine whether or not the individual has obtained protection by assigning P_j^k the value of a Bernoulli trial with parameter α_1 (the probability a recently vaccinated individual will be protected if challenged with smallpox, and refer to this as a vaccine success rate); recall that in general we denote the protection probability for a person in immune class i as α_i (see Table 5 for a summarization of the immune classes). If the individual is infected and in stage 1, then with probability α_1 they will be protected from disease; we then assume $P_j^k = 1$ and $G_j^k = 0$ (and the progression events that were associated with individual j are removed from the event list). Also, with probability $(1 - \alpha_1)\alpha^*$ the infection will be ameliorated: we compute S_j as a multinomial random variable of size 1 with parameters σ_1 (the vector of severity probabilities for recently vaccinated individuals). Finally, if the individual is infected in stage 2, it is too late for them to be protected from disease, but the infection may still be ameliorated with probability α^*: S_j^k is assigned the value of a multinomial random variable of size 1 with parameters σ_1. We always assume that σ_1 corresponds to a severity spectrum no more severe than for uninfected individuals (σ_0). We assume that the vaccine take is in the range 0.95–1 (defined to be the probability that a person develops a "Jennerian" reaction if the vaccine is administered). We assume that the degree of protection (the probability that a person in whom the take was successful will be protected) is 0.99–1 after the first week, and 0.5–0.99 during the first week, or prior to exposure, or if the vaccination is from several decades prior to infection. The amelioration

probability α^* is assumed to be in the range 0–1. These ranges were chosen to yield a large range of plausible values for sensitivity analysis.

Vaccine death events. As before, whenever a death event happens for person j at time t^k, all other events for person j become inactive, and $D_j^k = 1$, $S_j^k = 0$, and $G_j^k = 0$.

Full vaccine protection events. When an individual develops their fullest protection (the $\mathcal{F}_j$ occurs), then $Z_j^k = 0$ but $M_j = 3$ (full vaccine immunity). If $P_j^{k-1} = 0$, we assign P_j^k the value of a Bernoulli trial with probability α_3 (the probability of full vaccine protection, i.e. the vaccine success rate for individuals who have received the fullest protection).

New day begins. A event to begin a new day is always active, and the event time is always one day after the last new day event to occur. When a new day occurs, $K^k = K_r$ (the ring vaccination capacity is restored). The ring vaccination capacity K_r is chosen from the range 0–100 unless otherwise indicated.

The above specification of the state space, allowable events, event list, and transition rules constitutes a formal specification of the model [53]. The model was implemented in the C++ language and run on Linux workstations and on an Sun Solaris platform.

Partial rank correlation

We use the partial rank correlation coefficient [44, 45, 57] as a measure of the sensitivity of any model output with respect to any parameter, holding all other parameters constant. The (sample) partial correlation coefficient of variables W_1 and W_2 with the collection $X_1, \ldots, X_N$ held constant (with respect to $X_1, \ldots, X_N$) is defined to be the correlation of

the residuals of W_1 and W_2 after fitting these to $X_1, \ldots, X_N$, i.e. $\mathrm{cor}(W_1 - \hat{W}_1, W_2 - \hat{W}_2)$, where $\hat{W}_i = \mathbf{X}(\mathbf{X}^T\mathbf{X})^{-1}\mathbf{X}^T W_i$, $\mathbf{X}$ is the usual design matrix (the ith column of $\mathbf{X}$ is X_i), and $\mathrm{cor}(U_1, U_2)$ denotes the (Pearson) product-moment correlation between any quantities U_1 and U_2.

If $X_j^1, X_j^2, \ldots, X_j^i, \ldots, X_j^n$ are n observations for some variable X_j, a permutation k_i of $1, \ldots, n$ is a ranking of $X_j^1, \ldots, X_j^n$ (and k_i is a rank of X_j^i) if $X_j^{k_1} \leq X_j^{k_2} \leq \cdots \leq X_j^n$; if all the inequalities are strict, the rank is unique; otherwise, the rank is defined as the average of all the possible rank orders (i.e., the rank of "tied" observations is the average rank); for conciseness, $\mathrm{rank}(X_j)$ denotes the vector of ranks for the observations X_j $(X_j^1, \ldots, X_j^n)$. The partial rank correlation of W_1 and W_2 holding $X_1, \ldots, X_N$ constant is computed as the partial correlation coefficient of $\mathrm{rank}(W_1)$ and $\mathrm{rank}(W_2)$ with respect to $\mathrm{rank}(X_1), \ldots, \mathrm{rank}(X_N)$ (i.e., holding $\mathrm{rank}(X_1), \ldots, \mathrm{rank}(X_N)$ constant).

Table 1: **State of each individual** in the model.

Variable	Interpretation
S_j	Severity for individual j; values 0–5
G_j	Stage of smallpox for individual j; values 0–9 (see Figure 1 and Table 6)
Z_j	Indicator of very recent vaccination of person j;
	1 if person has been very recently vaccinated, 0 otherwise
V_j	Visit generation for individual j:
	0–diagnosed as a case when visited;
	1–visited as a non-case contact of a case;
	2–visited as a non-case contact of a non-case contact of a case
	3–unvisited;
M_j	Immunity status of person j
	0–no protection;
	1–partial protection due to very recent vaccination;
	2–partial protection due to vaccination in the distant past;
	3–fuller protection due to recent vaccination;
	4–fullest protection due to recovery from smallpox infection
P_j	Protection status; 1 if person j has protection against infection
A_j	Alert status; 1 if person j is alerted, 0 else
θ_j	Infection time
U_j	Undiagnosed indicator; 1 if individual j has ever been diagnosed, 0 otherwise
D_j	Dead indicator; 1 if person j is not alive

Table 2: **Smallpox stages**; see Figure 1.

State, G_j	Status	Duration (Ordinary Smallpox)
0	Never infected	-
1	Vaccine may prevent or moderate disease	0–4 days
2	Vaccine may moderate disease	0–3 days
3	Asymptomatic but too late for vaccination	0–11 days
4	Pre-eruptive symptoms, no enanthem	0–3 days
5	Pre-eruptive symptoms, enanthematous	0–1 day
6	Rash, period of maximum transmissibility	7 days
7	Rash, lowered transmissibility	7 days
8	Rash, scabbing; lowered transmissibility	7 days
9	Recovered, not infected	-

Table 3: **Active events.** In this table, we list all the (possible) events of the model, together with the state conditions under which the event is active (possible). For instance, a progression event $\mathcal{P}_j$ is active for person j whenever the smallpox status G_j for person j corresponds to active infection ($1 \leq G_j \leq 7$). For all events involving person j, the person j must be alive for the event to be active (possible), i.e. $D_j = 0$.

Event	Interpretation	Conditions under which event is active (possible)
$\mathcal{P}_j$	Progression	$1 \leq G_j \leq 7$, $D_j = 0$
$\mathcal{D}_j$	Death from smallpox	$6 \leq G_j \leq 8$ and $D_j = 0$ for $2 \leq S_j \leq 4$
		$G_j = 5$ and $D_j = 0$ for $S_j = 5$
$\mathcal{I}_j$	Infection	$G_j = 0$, $D_j = 0$ and $\sum_{j=1}^{N} I_j U_j > 0$
		(person is uninfected and susceptible;
		there is at least one undiagnosed infective)
$\mathcal{V}_j^0$	Visit to diagnose case	$3 \leq G_j \leq 7$, $D_j = 0$ (Person is symptomatic)
$\mathcal{V}_j^1$	Attempt to trace	$1\{V_j \geq 2\} \sum_{i=1}^{N} 1\{V_j = 1\} F_{ji} > 0$, $K > 0$, and $D_j = 0$
	contact of case	(person not visited as contact of case;
		can be traced from a case, capacity is present)
$\mathcal{V}_j^2$	Trace contact of contact	$1\{V_j = 3\} \sum_{i=1}^{N} 1\{V_j = 2\} F_{ji} > 0$, $K > 0$, and $D_j = 0$
	of case	(person not visited; can be traced from a contact)
$\mathcal{M}_j$	person j mass vaccinated	$M_j = 0$, $M = 1$, and $D_j = 0$
		(person not recently vaccinated, not had smallpox itself)
$\mathcal{C}_j$	Vaccine death for j	$Z_j = 1$ and $D_j = 0$ (person recently vaccinated)
$\mathcal{F}_j$	Full vaccine protection for j	$Z_j = 1$ and $D_j = 0$ (person recently vaccinated)
$\mathcal{N}$	Begin new day	always active

Table 4: **Parameter values**, part 1. The values used for Figure 3A are given in the column "Value"; where appropriate, the uncertainty analytic range is given in the column "Range".

Description	Symbol	Value	Range
Population Size	N	10000	-
Number initially infected (attack size)	A	10	-
Fraction vaccinated long ago	f	0.25	0–0.5
Ring vaccinations per day	K_r	-	-
Household size	H	4	1–10
Fraction with workplace/social contacts	w	1	0–1
Workplace/social group size	W	8	3–10
Mass vaccination to occur	M	0	-
Farthest contact to trace	τ	2	-
Infection hazard for close contacts	λ	5	0.5–10
Relative hazard for workplace/social contacts	h_2	1/3	0.1–1
Casual transmission rate	β	0.15	0–3
Delay in tracing household contacts	δ_1	1 day	1–2 days
Delay in tracing workplace/social contacts	δ_2	2 days	2–3 times δ_1
Vaccine take	ζ	0.95	0.95–1
Amelioration parameter	α^*	0.5	0–1
Vaccine success rate, $M_j = 1$ (for very recent vaccination)	α_1	2/3	0.5–0.99
Vaccine success rate, $M_j = 2$ (for vaccination prior to discontinuation of routine vaccination)	α_2	0.5	0.5–0.99
Vaccine success rate, $M_j = 3$ (full protection)	α_3	0.999	0.99–1
Time until full protection	D_f	7 days	-
Monitored diagnosis rate for previously asymptomatic contacts	ϕ	0.75 per day	0–1.5 per day
Relative diagnosis rate for previously asymptomatic contacts	f_1	1.5	1–2
Relative diagnosis rate after first diagnosed case	a_1	1.5	1–2
Vaccine mortality probability	v	10^{-6}	-
Vaccine mortality time	D_v	10 days	-
Probability of finding a household contact	ν_1	0.95	0.9–1
Probability of finding a workplace/social contact	ν_2	0.8	0.4–0.9
Probability of finding a casual contact		0	-

Table 5: **Parameter values** used in Figure 3A and other simulations, continued. Biomedical features of smallpox other than severity and progression; full details are in Appendix 1 and in Appendix 2 (Events), p. 48–51. The column labeled "Value" provides the value used in Figure 3A; the column labeled "Range" provides the uncertainty analysis range. Note 1: Assumed to be between 0.1 and 0.5 of the diagnosis rate for smallpox, stages 6–8 (severe, or ordinary/modified, as appropriate). Note 2: For the uncertainty analysis, we assumed slightly more rapid diagnosis due to the greater severity of symptoms; for Figure 3A, we explored the possibility that diagnosis of severe smallpox may take longer due to the less distinctive nature of the rash.

Description	Value	Range
Relative infectivity in modified type smallpox	0.5	0–1
Relative infectivity in mild smallpox	0.25	0–0.5
Relative infectivity, stage 4 relative to stage 5 (k)	0.2	0–1
Relative infectivity, stage 5 relative to stage 6 (k')	0.2	0–1
Relative infectivity, stages 7–8 relative to stage 6	0.5	0–1
Diagnosis rate in severe smallpox, stage 1-3	0	-
Diagnosis rate in severe smallpox, stage 4-5	0.3 per day	Note 1
Diagnosis rate in severe smallpox, stage 6-8	1.5 per day	0.75–2.5 per day<br>Note 2
Diagnosis rate in ordinary and modified smallpox, stage 1-3	0	-
Diagnosis rate in ordinary and modified smallpox, stage 4-5	0.5 per day	Note 1
Diagnosis rate in ordinary and modified smallpox, stage 6-8	1.25 per day	0.5–2 per day
Diagnosis rate in subclinical smallpox, all stages	0 per day	-
Hemorrhagic smallpox, stage 3, death probability	0.08	0.04–0.1
Hemorrhagic smallpox, stage 4, death probability	1	-
Flat smallpox, stage 6, death probability	2/3	0.5–0.8
Ordinary smallpox, stage 6, death probability	0.25	0.1–0.3
Modified-type smallpox, stage 6, death probability	0.01	0.0-0.03

Table 6: **Parameter values** used in Figure 3A and other simulations, continued. Probability of developing the indicated severity class of smallpox. The uncertainty analytic ranges are given on page 51.

Vaccine status	Severity class 1	2	3	4	5
Unexposed, unvaccinated	0.03	0.03	0.85	0.08	0.01
Vaccinated	0.05	0.25	0.62	0.03	0.05

Table 7: **Parameter values** used in Figure 3A and other simulations, continued. Progression delays through stages; the uncertainty analytic ranges are given under the subsection "Progression events" in Appendix 2, page 46.

Severity	Stage	Lower bound	Upper bound	Distribution
All	1	2.0	3.25	Uniform
All	2	2.25	3.75	Uniform
All	1–3	7	19	Scaled beta, see text
All	4	1.8	3.2	Uniform
Hemorrhagic	5	0	0.1	Uniform
All except hemorrhagic	5	0.95	1.25	Uniform
Flat, Ordinary, Mild	6	5.5	8	Uniform
Flat, Ordinary, Mild	7	5.5	7	Uniform
Flat, Ordinary, Mild	8	5.5	7	Uniform
Modified	6	4.75	6.75	Uniform
Modified	7	5.25	6.75	Uniform
Modified	8	5.25	6.75	Uniform
